# Comparative analysis of time-based and quadrat sampling in seasonal population dynamics of intermediate hosts of human schistosomes

**DOI:** 10.1371/journal.pntd.0007938

**Published:** 2019-12-20

**Authors:** Javier Perez-Saez, Théophile Mande, Dramane Zongo, Andrea Rinaldo

**Affiliations:** 1 Laboratory of Ecohydrology, Ecole Polytechnique Fédérale de Lausanne, Lausanne, Switzerland; 2 Départemente Biomédical et Santé publique, Institut de Recherche en Sciences de la Santé, Ouagadougou, Burkina Faso; 3 Dipartimento ICEA, Università di Padova, Padova, Italy; University of California Berkeley, UNITED STATES

## Abstract

**Background:**

Despite their importance for designing and evaluating schistosomiasis control trials, little attention in the literature has been dedicated to sampling protocols for the parasite’s snail intermediate hosts since their first development. We propose a comparative analysis of time-based and quadrat sampling protocols to quantify the seasonal variations in the abundance of these aquatic snail species of medical importance.

**Methodology/Principal findings:**

Snail populations were monitored during 42 consecutive months in three types of habitats (ephemeral pond, ephemeral river and permanent stream) in two sites covering different climatic zones in Burkina Faso. We employed both a widely used time-based protocol of 30min of systematic collection at a weekly interval, and a quadrat protocol of 8 replicates per sample at a monthly interval. The correspondence between the two protocols was evaluated using an ensemble of statistical models including linear and saturating-type functional forms as well as allowing for count zero-inflation. The quadrat protocol yielded on average a relative standard error of 40%, for a mean snail density of 16.7 snails/m^2^ and index of dispersion of 1.51. Both protocols yielded similar seasonal patterns in snail abundance, confirming the asynchrony between permanent and ephemeral habitats with respect to the country’s seasonal rainfall patterns. Formal model comparison of the link between time vs. quadrat counts showed strong support of saturation for the latter and measurement zero-inflation, providing important evidence for the presence of density feedbacks in the snail’s population dynamics, as well as for spatial clustering.

**Conclusions/Significance:**

In addition to the agreement with the time-based method, quadrat sampling provided insight into snail population dynamics and comparable density estimates across sites. The re-evaluation of these “traditional” sampling protocols, as well as the correspondence between their outputs, is of practical importance for the design and evaluation of schistosomiasis control trials.

## Author summary

The ongoing paradigm shift from schistosomiasis morbidity control to elimination will require addressing multiple facets of the disease’s transmission cycle, including control of the snail obligate intermediate hosts of the parasite. In this context the choice of sampling protocol to monitor and understand population dynamics of aquatic snails has practical implications, in particular for studying the strong seasonal fluctuations in their abundance. However, comparative analyses of alternative methods have seldom been carried out since their development in the 1960s-1970s. Here we compare a widely used time-based protocol with a less commonly employed quadrat protocol. Data collected during 3.5 years in three different habitat types in Burkina Faso (West Africa) suitable for the quadrat method show that both protocols agree in the detection of abundance seasonality. By using statistical models to link the outputs of the two methods we also highlight a saturation of quadrat absolute density estimates as well as zero-inflation in the counts. These results provide insight into the snail’s population dynamics with respect to time-based sampling by supporting the presence of density feedbacks as well as small-scale spatial clustering. This work fosters the use of quadrat sampling in other contexts and the comparison of results with other sampling protocols.

## Introduction

Advances in the control of neglected tropical diseases (NTDs) hinge on the numerous social-ecological drivers that support their complex transmission dynamics [1]. Among these drivers the ecology of intermediate hosts and vectors is a common trademark of the lifecycles of the parasites causing NTDs [2]. The ability to gain information on the ecology of these species is therefore paramount for control programs to identify transmission sites, but also to tailor intervention strategies to specific settings [3]. In this respect, the choice and design of ecological sampling protocols for the monitoring of intermediate host and vector populations are crucial for control programs aiming at elimination by determining the type and quality of information gathered on these species’ population dynamics [3].

Schistosomiasis is a striking example of the importance and challenges of ecological monitoring in NTD control strategies. Caused by flatworms of genus *Schistosoma*, the disease affects more than 140 million people worldwide [4]. Schistosomes require specific species of aquatic or amphibious snails to complete the asexual reproduction phase of their lifecycles [5]. There has been increasing attention to schistosomes’ snail obligate intermediate hosts with the recent paradigm shift from morbidity control to elimination through transmission interruption following the World Health Assembly 2012 resolution 65.21 on schistosomiasis elimination [6, 7]. The analysis of past successes in disease elimination have highlighted the key role of snail control in reaching transmission interruption [8–10]. Snail control can be achieved through habitat modification, chemical or plant-based molluscicides [11], or by biological control with snail predators such as fish [12] or prawns [13].

The importance of snail monitoring and control has been illustrated by the experience of the national schistosomiasis control program in China which has implemented an integrated approach since 2004 [14]. Therein, the systematic sampling of *Oncomelania hupensis*, the amphibious snail host of *Schistosoma japonicum*, using snail traps enabled to evaluate the effect of habitat modification and mollusciding campaigns in particular in places where transmission was close to interruption [15]. As China approaches the elimination phase recent focus on snail monitoring has been on novel methods for the detection of infection in snails as opposed to measuring variations in snail density [16]. Examples of the importance of snail monitoring and control in other countries include the control programs of Iran, Tunisia, Morocco and Egypt [8, 9].

In spite of the advances in schistosomiasis control in China, more than 80% of people still affected by schistosomiasis live in sub-Saharan Africa (SSA) [4]. Mass drug administration (MDA) campaigns have successfully reduced morbidity in most targeted endemic countries with a coverage of almost 70% of school aged children in 2017 [17]. These gains underpin a shift towards transmission interruption [7]. A key difference between the two socio-ecological transmission settings is the ecology of the snail species that serve as intermediate hosts of the main human schistosome species in the SSA, *Schistosoma mansoni* and *S. haematobium*, which pose challenges for transferring the Chinese experience to SSA [18].

The snail species which host them are respectively from the *Biomphalaria* and *Bulinus* genera [5, 19]. As opposed to *O. hupensis*, both genera are aquatic although they are able to survive long periods of desiccation through aestivation allowing them to occupy a wide diversity of permanent and temporary natural or man-made aquatic habitats [19]. Moreover these species are hermaphroditic, i.e. are able to self-fertilize [20, 21], which enables them to very rapidly re-colonize habitats targeted by control measures. Finally, the population dynamics of these snail species are characterized by strong annual fluctuations driven by site-dependent climatic and hydrological factors, in particular in seasonal climates [22–24].

A striking example of these strong seasonal fluctuations are dry areas such as the Sahelian climatic zone where hydrological conditions impose narrow windows of water presence during which snail populations can reproduce in areas such as temporary ponds and lakes as well as ephemeral streams [25]. These habitats typically host *Bulinus* spp. such as *B. senegalensis* and *B. truncatus* which have very high reproduction rates and long aestivation potential (6 months or more). The very fast colonization of habitats following the first seasonal rains typically coincides with intense human-water contact thus favouring disease transmission [26]. Beyond presence or absence of water, variations in water flow velocity in less arid climates pose important constraints on snail ecology with velocities larger than approximately 0.4m/s being unsuitable for their presence [19]. In small streams fast variations in discharge may therefore lead to spatial heterogeneities in snail population densities along the shores [27]. Strongly seasonal environments are therefore particularly challenging for the implementation of sampling protocols due to the variations in the hydrological conditions supporting snail population dynamics [28].

The specificity of the ecology of the snail intermediate hosts of schistosomes in SSA is of great relevance from the schistosomiasis control perspective. On a practical level, seasonal variations in snail populations enable the timing of chemotherapy and snail control measures due to context-specific parasite transmission windows during the year [28–30]. Beyond the requirement of snail host presence for transmission to occur, incidence has been more consistently linked to the density of infected snails rather than total snail abundance [31, 32]. However the temporal variation of infected and total snail densities can be linked in some cases when accounting for the duration of pre-patency during which snails are infected but not yet shedding cercariae, schistosome larvae that infect humans [33].

On a more fundamental level, the existence of negative density feedbacks in snail population dynamics, i.e., the limitation of reproduction or survival due to crowding, have direct implications for the implementation of control strategies. First and foremost density feedbacks determine the strength of populations rebounds that can be expected after treatment, also called compensatory density feedbacks as highlighted for *O. hupensis* in different types of habitats in China [34]. In addition, the reduction of snail abundance in the presence of negative density feedback could lead in certain cases to a net increase in cercarial production by the remaining infected snails due to the resulting per capita increase in limited resources [35]. This nonlinear link between snail density and cercariogenesis could thus potentially undermine the aim of reducing transmission through snail control [36]. It is therefore of practical importance for ecological sampling protocols designed to monitor the population dynamics of snail intermediate hosts of schistosomes to accurately detect seasonal variations in abundance, but also to provide insight into the underlying ecological dynamics with particular emphasis on density feedbacks.

Most snail population sampling protocols used today were established by research efforts between the 1950’s and the 1970’s fostered by a strong focus on transmission control through snail population management [10, 37, 38]. Recent quantitative estimates of snail abundance in the context of schistosomiasis control trials in SSA have principally relied on methods involving active habitat sampling principally using time-based protocols, as opposed to passive methods such as snail traps [31, 39]. Time-based protocols involve systematically collecting snails for a limited time duration within a pre-defined area, presenting the advantage of being relatively cheap, rapid and easily implemented [40]. However these protocols can only produce relative density estimates due to collector-dependent variability in sampling efficiency as well as differences in habitat type and geometry [37]. Data collected using this method are therefore limited to the quantification of seasonal population variations but are not directly comparable in terms of snail abundance across sites and studies. Moreover they do not give direct information on the presence and strength of density feedbacks, although these can be inferred using ecological modelling with multiannual data [24].

Absolute density protocols on the other hand can produce data that are comparable across sites and provide additional insight into snail population dynamics. Despite their advantages, absolute snails densities have been used in a limited number of studies involving either mark-recapture protocols [22, 23], exhaustive habitat sampling [41], or quadrat protocols [42, 43] (reviewed in Table 1). Of these methods, exhaustive sampling is only possible for small waterbodies (≈ 10m^2^). Mark-recapture methods present the advantage of providing estimates of recruitment and mortality. However this method is particularly labor-intensive, and requires the collection and processing of a large amount of snails (≈ 1000) to obtain reasonable population estimates [44]. Quadrat sampling on the other hand provides absolute density estimates with simpler and less labor-intensive protocols. It has however seldom been used in comparison to mark-recapture protocols possibly due to the practical difficulty of applying it to habitats with water depths exceeding the order of centimeters. Nevertheless quadrat sampling is applicable in many transmission settings, in particular in habitats with relatively low vegetation cover. Moreover quadrat sampling protocols are simpler and have lower human labor requirements in comparison to mark-recapture methods which is an important factor for schistosomiasis national control programs with limited resources.

**Table 1:** Literature review of absolute density estimates of the snail intermediate host species of schitosomiasis in sub-Saharan Africa. Studies are subdivided by sampling method. Details are given for the size of the habitat for exhaustive sampling and quadrat size and number of replicates per sample (in parentheses) for quadrat sampling. Standard errors of the mean where computed from the reported confidence intervals assuming they were given for the 95% interval.

	Country	Sampling area	Species	Habitat	Density [snails/m^2^] (SE)	ref.
**Mark-recapture**						
	Nigeria	-	*Biomphalaria pfeifferi*	Isolated pool	51 (-)	[42]
	Nigeria	-	*B. pfeifferi*	Reservoir	16 (-)	[42]
	Zimbabwe	-	*B. pfeifferi*	Rivers	2.4-98 (-)^1^	[23]
	Zimbabwe	-	*Bulinus globosus*	Rivers	6-230 (-)^2^	[22]
**Exhaustive**						
	Tanzania	11.75 m2	*B. pfeifferi*	Isolated pool	26.6 (0.87)	[41]
**Quadrats**						
	Nigeria	15x15cm (-)	*B. pfeifferi*	Reservoir	83 (11.9)	[42]
	Nigeria	15x15cm (-)	*Bulinus truncatus*	Reservoir	2.4 (0.59)	[42]
	DR Congo	25x25cm (15)	*B. pfeifferi*	River	14.6-150.1 (-)^3^	[43]

^1,2^ Range of peak density values from 2 and 5 different rivers respectively

^3^ Minimum-maximum range of observed values in one river

As the ongoing paradigm shift in schistosomiasis control brings focus back on snail monitoring and control, a direct comparison between commonly used time-based sampling protocols and absolute density methods can help pose a basis for choosing among them as part of an integrated social-ecological approach. We here focus on the comparison between this widely used protocol and quadrat sampling. The objectives of the study follow from the requirements of sampling protocols to quantify seasonal snail population variations as well as detecting density feedbacks: i) the definition of design requirements of a quadrat sampling scheme in terms of accuracy and precision, ii) the evaluation of the correspondence in the seasonal variations of snail abundances between time-based and quadrat methods, and iii) establishing a statistical link between the outputs of the two sampling strategies in the different types of habitats. Regarding the latter point, our previous analysis inferred the presence of density feedbacks in snail population dynamics across habitats [24]. Our hypothesis in this study is therefore that absolute snail densities should reflect habitat-dependent carrying capacities. Our second working hypothesis is that time-based counts present a higher temporal variability than quadrat-based counts. Taken together, these lead to the hypothesis of a saturating-type relation between time-based relative density and absolute density estimates.

## Materials and methods

### Field sites

**Fig 1:**
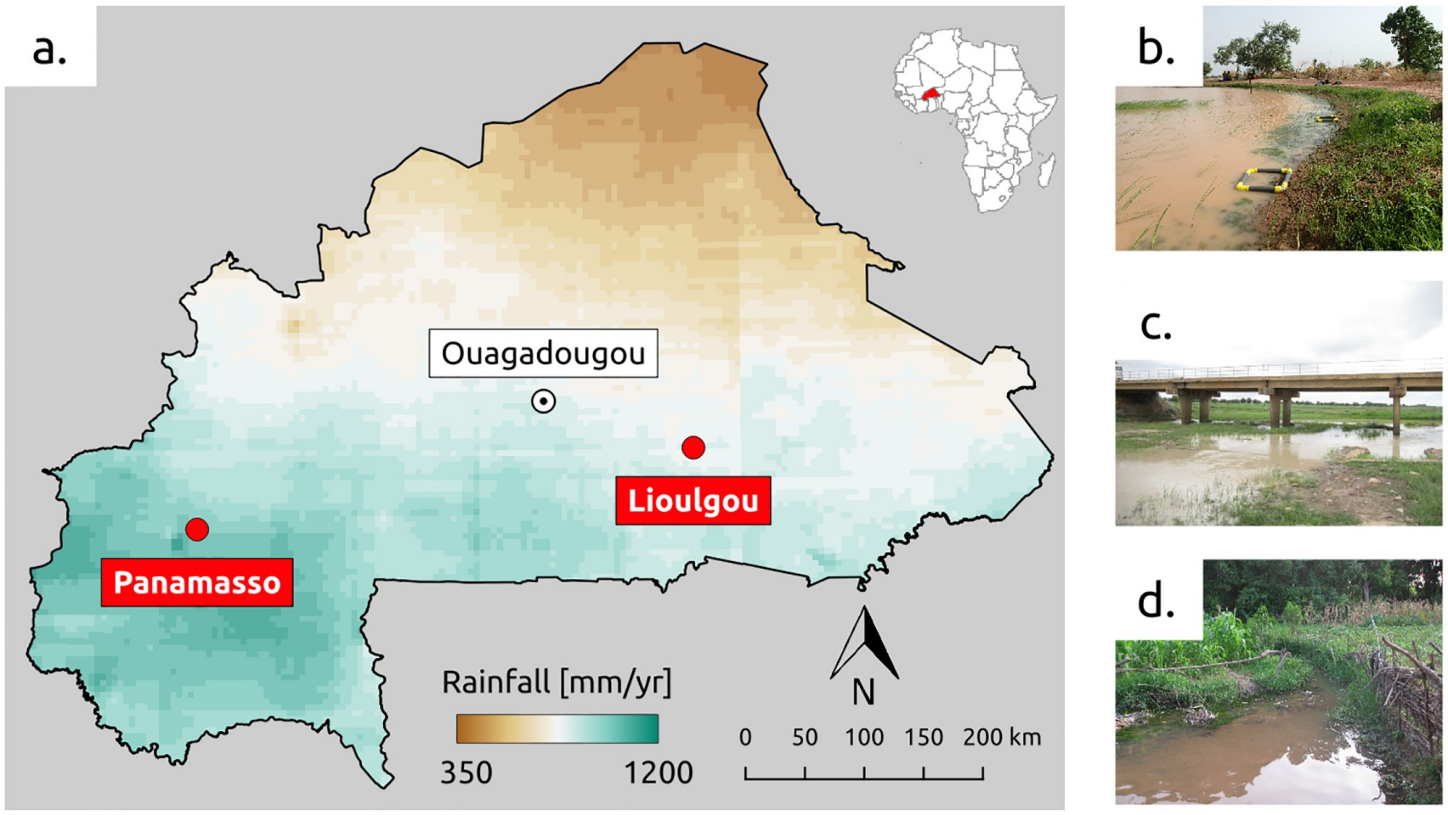
Situation map of field sites and habitats. a. Burkina Faso with its capital (Ouagadougou) and the location of the two field sites (in red) along the precipitation gradient (2014-2017 average, CHIRPS data [51]). The map was produced with QGIS (version 3.6) with country boundaries provided by GADM (version 3.4). Sampled habitats in Lioulgou included an ephemeral pond (b) and a river (c), and a permanent stream in Panamasso (d).

The malacological data was collected in two field sites located in Burkina Faso (West Africa) along the South-North climatic gradient between the Sudanian and Sahelian climatic regions (Fig 1a, detailed description given in [24]). Annual rainfall in these climatic zones ranges from 350mm to 1100mm per year, and presents a marked seasonal variation with most precipitation occurring during the rainy season between July and September and only very limited precipitation events the rest of the year (Fig 1) [45]. Both intestinal and uro-genital schistosomiasis are present in the country [46]. MDA campaigns initiated by the Schistosomiasis Control Initiative since 2005 have successfully brought down prevalence in most regions with a national mean prevalence of around 5% in school aged children in 2013 [47]. Reductions in transmission have however been less successful in the Northern and Southwestern parts of the country with persistent hot spots of both forms of the disease [48, 49]. In the face of this evolution the Ministry of Health has set elimination as the goal, although the strategy will be principally based on chemotherapy without snail control [47].

Previous malacological studies in the country identified various species of *Bulinus* as well as *Biomphalaria pfeifferi* colonizing both natural and man-made habitats across the country’s climatic gradient [25]. The probability of occurrence of *B. pfeifferi*, an intermediate host of *S. mansoni*, has been linked to the geography of intestinal schistosomiasis, specifically in the Southwestern part of the country [46, 50]. Multiple species of *Bulinus* have been reported both in temporary and permanent aquatic environments including ponds, lakes, reservoirs, rivers and irrigation canals [25]. The association between snail density and schistosome infection prevalence in humans has not been studied across different climatic zones in the country [26].

The choice of the experimental sites aimed at covering different climatic conditions along the precipitation gradient, the habitat preferences of the snail intermediate hosts [24, 46], and logistic constraints including site accessibility, travel time, and total available budget for the study. Moreover both sites are part of the Ministry of Health’s schistosomiasis sentinel sites in the country [47]. Habitat selection for sampling within each site was guided by prior knowledge of important transmission sites identified by the National Schistosomiasis Control program. Among transmission sites, a limited selection was retained to cover different types of habitats due to resource constraints. The first site was located in the village of Lioulgou (12°00′38”N, 0°21′48”W) in a floodplain in the Sudano-Sahelian climatic region approximately 150km East of the country’s capital Ouagadougou. Two habitats were monitored, a pond (Fig 1b) and a river (Fig 1c), both of which dry out around 7 months per year. The size of the waterbodies varies strongly during the season, with maximal extents of 10m width for the river and 3’000 m^2^ for the pond. The second site was located in the village of Panamasso (11°23′23”N, 4°12′02”W) in the Southwestern part of the country about 300km South-West of Ouagadougou. This region is characterized by a Sudanian climate and permanent river flow regime conditions [50]. The monitored habitat consisted of a small 2m wide permanent stream adjacent to the village (Fig 1d). This stream constitutes the main water source for domestic and recreational activities in the village (laundry, dish washing, bathing).

### Sampling protocols

Malacological data collection was done during 42 months from July 2014 to December 2017. Sampling was undertaken by field technicians trained by our campaign management. Training was provided during one week before the start of the collection period in July 2014, and feedback was given by bi-weekly contacts (phone calls) and regular on-site visits during the entire study period up to December 2017. Biological samples were sent every 3 months back to the Microbiology and Biotechnology Laboratory of the Institut International d’Ingénierie de l’Eau et de l’Environnement (2iE) in Ouagadougou for recounting and species identification using standard identification keys [19]. For logistical reasons sampling in Lioulgou (pond and river habtitats) could not be done in 2018, and neither the quadrat sampling in Panamasso between June and October of 2016.

#### Time-based sampling

A time-based snail sampling technique was employed analogously to the one previously used in malacological studies in the country [52], and to the one typically employed in recent schistosomiasis control trials in SSA [31, 39]. The sampling protocol consisted of systematically scooping pre-defined areas of the investigated habitats for 30 minutes using a 2mm metal mesh where water depths permitted it [40]. In the very shallow sections of the habitats such as the shores of the pond and the water access point of the small stream, snail collection was made by hand-investigation of available snail supports (vegetation, debris, etc…) [53]. Time-based sampling was done at a weekly interval in both field sites. The time of the day the sampling took place was recorded, which was systematically done in the morning between 8AM and 9AM. The number of collected snails and sampling metadata were recorded in dedicated sampling sheets. *Bulinus* spp. or *Biomphalaria* spp. specimens were stored in dated collection cups in a mixture of clear well water and (90%) alcohol.

#### Quadrat sampling

Absolute snail density estimates were obtained at a monthly interval. We retained a size of 30x30cm quadrats. Previous studies that employed quadrat sampling referenced in Table 1 did not provide justifications for the choice of the number of quadrat replicates per sampling. Important considerations for this choice include effort constraints in terms of the time required for exhaustive counts in each quadrat on one hand, and the precision of the mean density estimate on the other [54]. We here chose to maximize the marginal gain in terms of the relative standard error on the estimate of mean density from each additional replicate considering limits in the overall sampling effort. The relative standard error of the mean *SE*/*μ* reads:

$$\begin{array}{c} \frac{SE}{\mu }=\frac{\sigma }{\sqrt{n}\mu }, \end{array}$$ (1)

where *n* is the number of samples taken. A level *SE*/*μ* = *α* is typically set to determine the number of required samples based on *a priori* knowledge on the spatial mean and variance of the number of snails of the investigated species per quadrat. For quadrat sampling the only available data report a relative standard error of 14.3% and 24.6% for *B. pfeifferi* and *B. truncatus* respectively with an unknown number of samples [42]. Estimates for *μ* from other studies are reported in Table 1, however prior information on *σ* was not available. To provide a working hypothesis to overcome this limitation, snails were assumed to have a clustered spatial distribution at the micro-habitat level, thus yielding over-dispersed quadrat counts. The degree of over-dispersion can be quantified by the index of dispersion relating the variance of the counts to their mean $I_{D}=\frac{\sigma^{2}}{\mu }$, where *I*_*D*_ > 1 indicates overdispersion. The only study quantifying the spatial dispersion of snail intermediate hosts of human schistosomes found in the literature gave monthly values of *I*_*D*_ of 1.8-2.7 for *Bulinus globosus* and 2.1-8.6 for *Biomphalaria pfeifferi* measured along consecutive river stretches of 40m length in Zimbabwe [27]. Given a known value for *I*_*D*_, the relative standard error can then be expressed as:

$$\begin{array}{c} \frac{SE}{\mu }=\sqrt{\frac{I_{D}}{n\mu }}, \end{array}$$

and the number of replicates to obtain a relative standard error level *α* as: *n* = *I*_*D*_/(*α*^2^*μ*). Assuming a density equal to the mean of the observed densities report in Table 1 of ≈ 67 snails/m^2^ (*μ* ≈ 6 snails/30x30cm quadrat), and snail spatial overdispersion in the same order of magnitude as the one observed in [41] (*I*_*D*_ = 2), the number of replicates for a given relative standard error level was *n* ≈ 1/(3*α*^2^). Taking into consideration the quadratic relationship between gains in precision and number of replicates as well as effort constraints for the field technicians after initial field trials in the field sites, the number of replicates was set to 8, which corresponded to a relative standard error of *SE*/*μ* ≈ 20%. In retrospect a simulation analysis of the quadrat sampling protocol based on the re-analysis of the data in [41] supported the choice of a 30x30cm quadrat with 8 replicates in terms of expected relative standard error (S1 Appendix). In fact, the size of the quadrats were in line with the microhabitat size of *B. pfeifferi* [41] (S1 Appendix). Moreover the value of the index of dispersion of 2 we used in the study design based on the literature fell within the 95% CI for the same sampling scheme setup, although the mean value was of 3.6 (S1 Appendix). In each habitat the quadrats were positioned at random where water was less than 20cm deep (approximately 1m from the shore in the pond, between 50cm and 1m for the river and streams), which corresponds to the observed microhabitat range for *B. pfeifferi* [41] assumed to be similar for *Bulinus* species found in Burkina Faso [25]. The random positioning of quadrats was determined at each sampling date by the field technicians by starting at one end of the sampling site (pre-defined for the time-based protocol). The randomization procedure consisted in standing on the shoreline and sequentially throwing the quadrats in a direction parallel to the shoreline aiming so that it fell between 2 and 5m of the standing place (end of the site or preceding quadrat), and within 50cm shoreline in the water. If the latter was not the case the field technician would reposition by hand within 50cm and 1m of the shoreline depending on the habitat. The time of the day the sampling took place was also recorded, which was done in the morning between 8AM and 11AM.

### Ethics statement

The collection of wild snail specimens did not require specific permits since neither snail genera are protected nor endangered species.

### Statistical analysis

The link between quadrat counts and time-based measures of relative abundance was explored using Poisson-type regression models for count data. To test the hypothesis of saturating quadrat-based counts with respect to time-based estimates we fitted for each habitat both linear and saturating-type functions of the monthly mean time-based measure of relative abundance to the corresponding quadrat counts using generalized linear models (GLM). Measurement variability of quadrat count data was modelled using Poisson-type likelihood functions, including the Poisson and Negative Binomial distributions as well as their zero-inflated and hurdle counterparts [55, 56]. Zero-inflated and hurdle models allow for the extra-occurrence of zeros in the quadrat count data. The parameters governing the zero-occurrence (zero-inflation for the former and the probability of zero for the latter) could also be linked to relative abundance measures, and were therefore incorporated either as a constant or as an inverse-sigmoid function of mean monthly time-based abundance. Details for the transfer functions between time-based means and quadrat counts, the measurement models as well as the expression of zero-occurrence parameters are given in Table 2. We call transfer functions the different types of functional forms to link the time-based and quadrat counts. All combination of these three model components were tested by finding the maximum-likelihood estimates (MLE) of the parameters using standard optimization software in R (version 3.4.4) [57]. Model selection was done using the compensated Akaike Information Criterion (AICc) which balances the quality of fit in terms of log-likelihood and the complexity of the model in terms of the number of parameters [58]. Relative support for each model was then determined using AIC weights $w_{i}=\frac{e^{-0.5\Delta {\text{AIC}}_{c,i}}}{\sum_{j}e^{-0.5\Delta {\text{AIC}}_{c,j}}}$, where ΔAIC_*c*_ denotes the difference between the AIC_c_ of model *i* and the minimum AIC_c_ score among all tested models [58]. Support for each model was computed in terms of model probabilities by summing the AIC weights for each model formulation. These represent the probability that a given model is the one that generated the data. Ratios of model probabtilities given the relative support of one model formulations [58]. AIC weights were also used to compute the unconditional variance of model parameters in the 95% model confidence set [58]. Simulations of the relationship between quadrat and time-based were then performed using the credible model set (S1 Table) for each species-habitat configuration. A total of 5000 simulations were performed for a range of time-based counts between 0 and 125 snails/30min, with a random selection of models based on their corresponding AIC weight. All the data presented in this work and the code used to produce the results are available on Dryad.

**Table 2:** Details of model structures. Equations are given for the alternatives of transfer function, measurement model and zero-occurrence parameter. All combinations of the three components were tested. The number of free parameters in each equation is denoted *n*.

Model	Equation	*n*
**Transfer function** λ = *f*(*x*)		
Linear	*a* + *bx*	2
Mikealis-Menten	$\frac{ax}{1+bx}$	2
monomolecular	*a*(1 − exp(− *bx*))	2
Logistic	$\frac{a}{1-\exp(b-cx)}$	3
Gompertz	*a* exp(− exp(*b* − *cx*))	3
**Measurement model** *P*(*Y* = *k*|λ)		
Poisson (*Poiss*)	$\frac{\exp\left(-\lambda \right)\lambda^{k}}{k!}$	0
Negative-Binomial (*NB*)	$\frac{\Gamma (r+k)}{k!\Gamma (r)}{\left(\frac{r}{r+\lambda }\right)}^{r}{\left(\frac{\lambda }{r+\lambda }\right)}^{k}$	1
Zero-Inflated Poisson	$\left\{ \begin{array}{cc} \sigma +(1-\sigma )\;\text{exp}(-\lambda ) & \;\text{if}\;k=0\\ \left(1-\sigma )Poiss(k\right) & \;\text{if}\;k>0 \end{array}\right.$	1
Zero-Inflated Negative binomial	$\left\{ \begin{array}{cc} \sigma +\left(1-\sigma \right){\left(\frac{r}{r+\lambda }\right)}^{r} & \;\text{if}\;k=0\\ \left(1-\sigma )NB(k\right) & \;\text{if}\;k>0 \end{array}\right.$	2
Poisson Hurdle	$\left\{ \begin{array}{cc} \sigma & \;\text{if}\;k=0\\ \left(1-\sigma \right)Poiss\left(k\right){\left(1-\exp\left(-\lambda \right)\right)}^{-1} & \;\text{if}\;k>0 \end{array}\right.$	1
Negative Binomial Hurdle	$\left\{ \begin{array}{cc} \sigma & \;\text{if}\;k=0\\ \left(1-\sigma \right)NB\left(k\right){\left(1-{\left(\frac{r}{r+\lambda }\right)}^{r}\right)}^{-1} & \;\text{if}\;k>0 \end{array}\right.$	2
**Zero-occurrence** *σ* = *g*(*x*)		
Constant	*σ*	1
Logistic	$1-\frac{1}{1-\exp(a-bx)}$	2

## Results

### Performance of the quadrat sampling scheme

**Fig 2:**
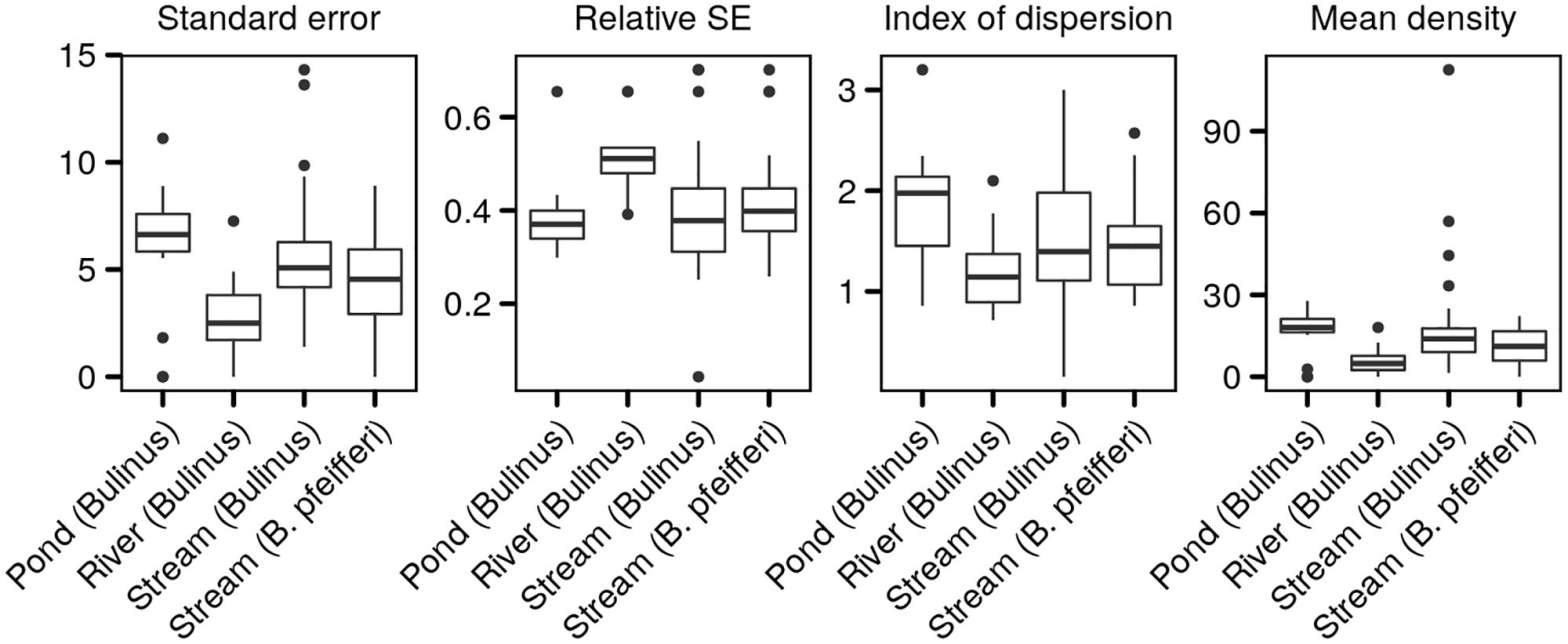
Quadrat sampling scheme performance. Reported are the distribution of standard errors ($SE=\sigma /\sqrt{n}\left[{\text{snails}}^{\frac{1}{2}}/\text{m}\right]$), relative standard errors (*SE*/*μ* [-]), the index of dispersion (*I*_*D*_ = *σ*^2^/*μ* [-]) and the mean density (*μ* [snails/m^2^]) by habitat and intermediate host species.

The quadrat sampling scheme yielded an average standard error on the mean of 0.46 snails/quadrat (5.22 snails/m^2^) with differences among habitat and species (Fig 2). Standard errors were the highest for *Bulinus* spp. in the temporary pond, and of similar magnitude for both snail genera in the permanent stream. The scheme performance in terms of relative standard error was inferior to the 20% aimed by our study design, with an average relative error of 41.8% across sites and species. This poorer performance reflects the much lower value of the mean number of snails per quadrat observed in our fieldsites (average across sites, species and months of 1.5 snails/quadrat, 16.7 snails/m^2^, as opposed to the 67 snails/m^2^ used in the study design) despite lower values of the index of dispersion (1.51 on average) than the value of 2 we assumed based on the literature as well as the one obtained by sampling simulation using 30x30cm quadrats on the data from the supplementary study of the dataset from Tanzania [41] (average value of 3.1, see S1 Appendix). However, the average standard error value is close to the one obtained using the latter simulation scheme (average of 35% see S1 Appendix).

### Population variations

**Fig 3:**
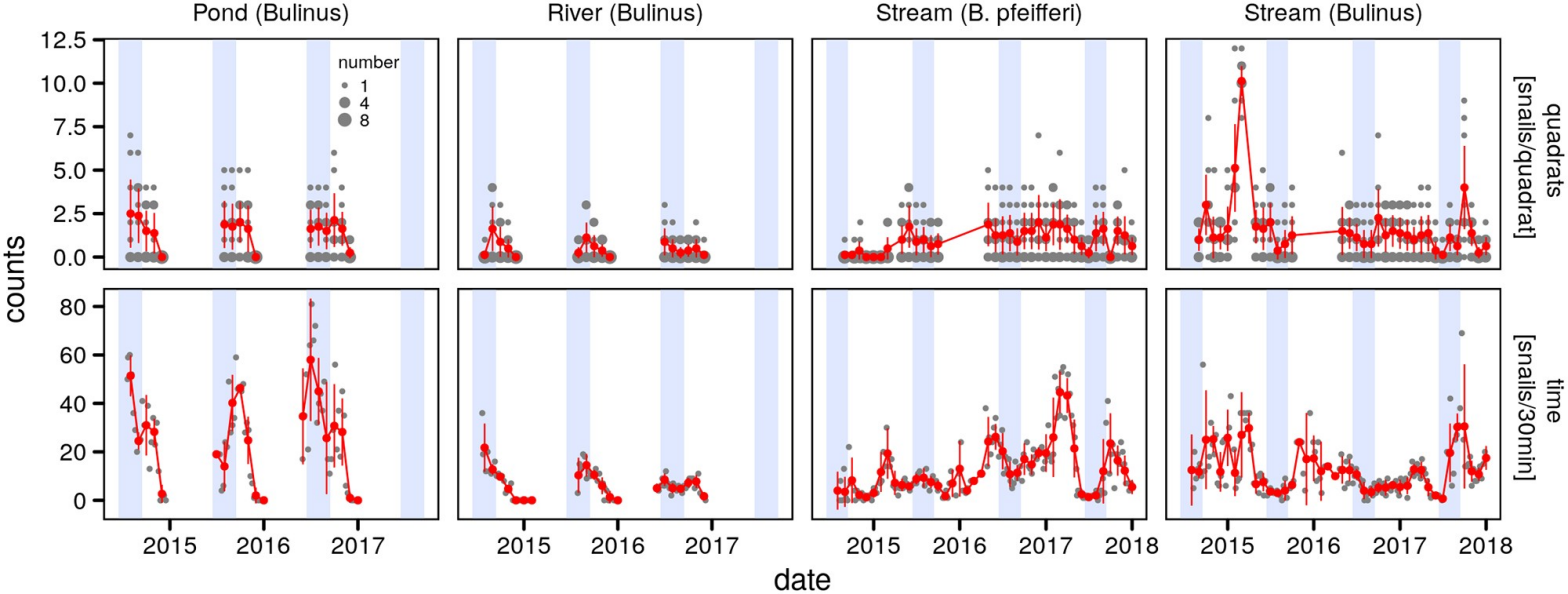
Ecological monitoring data of intermediate hosts of schistosomiasis in Burkina Faso. Data was collected in three different habitats in two distinct sites, one in the Sudano-Sahelian climatic zone (pond and river, village of Lioulgou), and one in the Sudanian climatic zone (stream, village of Panamasso). Data are presented in terms of the number of observed counts (grey points) by intermediate host genera for the quadrat (top row) and time-based method (bottom row) protocols with blue rectangles indicating timing of the rainy season (July-September). Monthly means (red points) for the quadrat method consist of the average of the 8 quadrat replicates on the sampling date, and the mean of the weekly time-based counts grouped to the closest month start, along with their 95% CI (red errorbars, mean ±1.96 SE). Note that quadrat data in the months of June-October of 2016 were not collected in the permanent stream in Panamasso due to logistical constraints.

The main *Bulinus* species that were collected were *B. truncatus* in the ephemeral stream in Lioulgou, *B. senegalensis* in the temporary pond in Lioulgou, and *B. globosus* in the perennial stream in Panamasso. *Biomphalaria pfeifferi*, the intermediate hosts of *Schistosoma mansoni* were only found in the latter. Snail population dynamics presented strong seasonal fluctuations both in the permanent and ephemeral habitats (Fig 3). As it had been previously observed for Burkina Faso [52, 59, 60], snail abundance in the ephemeral habitats was largest during the rainy season (July-September) and rapidly decreased with the disappearance of habitat into the winter season. The population dynamics of *Bulinus* spp. and *Biomphalaria pfeifferi* in the permanent stream in Panamasso were characterized by year-round presence in contrast to the ephemeral habitats.

The overlap of the mean monthly counts by day of the year confirms the similarity of seasonal variations detected by the two methods with the time-based sampling scheme presenting stronger variations (S1 Fig). For the ephemeral habitats (pond and river) both methods yield a sharp rise at the onset of the rainy season in June-July and the collapse of snail densities by December which corresponds to the drying out of the waterbodies. Interestingly both methods reveal lower population densities of *Bulinus* spp. in the ephemeral river in 2016. The seasonal trend for *Bulinus* spp. in the permanent stream was also captured in a similar manner by both methods with seasonal peaks in October-November and again in February-March. The time-based data presented stronger variability than the quadrat method as measured by the coefficients of variation (CV) (S1 Fig). One notable exception is *Bulinus* spp. in the stream for which the CV was higher for the mean of the quadrat samples, possibly driven by the very high density measured in February-March 2015 (Fig 3). In the case of *B. pfeifferi* the strongest seasonal variation was observed with the time-based sampling in 2016 for which the quadrat sampling only produced a weak signal.

**Fig 4:**
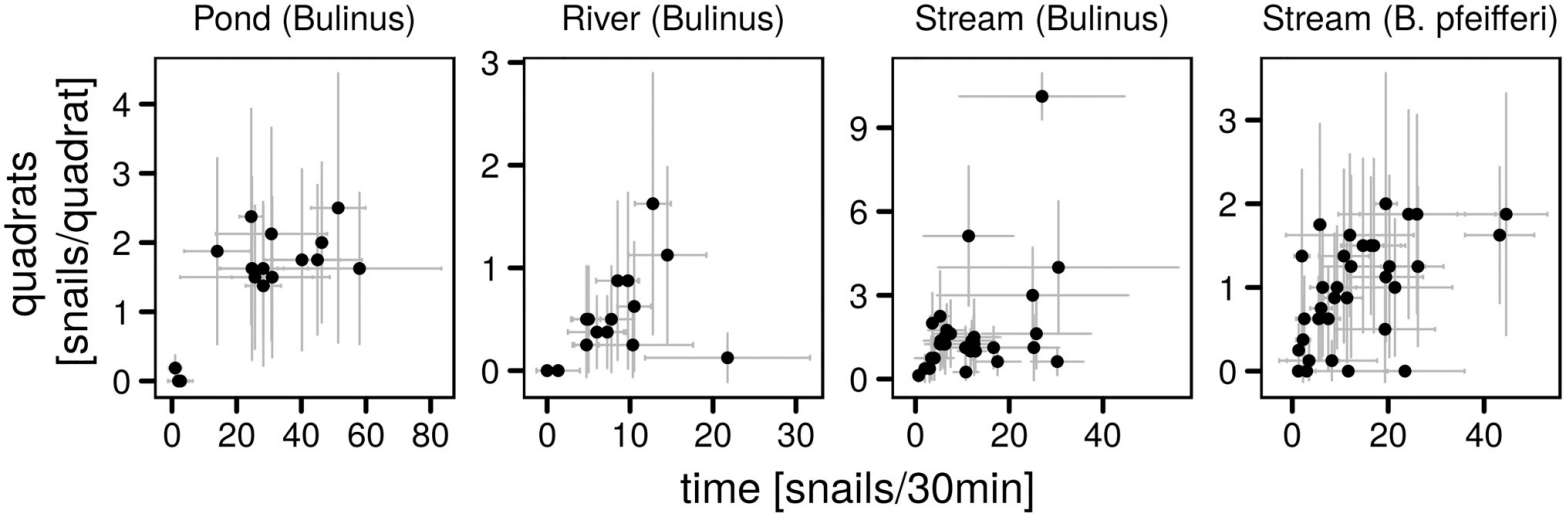
Comparison of monthly mean densities from the quadrat and time-based sampling schemes. Horizontal and vertical errorbars give the 95% CI of the time-based and quadrat-based means respectively.

A direct comparison of average densities estimated with the two sampling strategies further confirms the overall pattern of a correspondence between the methods but with the time-based samples presenting stronger variations than the quadrat absolute density estimates (Fig 4). The comparison suggests a stronger linear correspondence between the two methods at low time-based snail relative densities (< 20 snails/30min), with the quadrat absolute density estimates reaching a plateau at high time-based densities. This pattern is particularly visible for *Bulinus* spp. in the pond and for *B. pfeifferi* in the permanent stream. On the other hand the low *Bulinus* spp. densities in the ephemeral river do not suggest the existence of a plateau within the range of observed densities. The exceptionally high density of *Bulinus* spp. in the permanent stream also seemed to support the absence of a plateau in snail density. The non-linear correspondence between the sampling methods is supported by the analysis of Pearson and Spearman correlation coefficients as a function of the considered range of time-based densities (S2 Fig). The strongest significant Pearson correlations are observed for time-based densities < 20 snails/30min for *Bulinus* spp. in all three habitats, whereas the rank-correlation is strongest when considering wider ranges of time-based estimates. For *B. pfeifferi* both correlations are only significant above ≈ 15 snails/30min and have their maximum around 20 snails/30min. The differences between the two correlation indices therefore suggests a non-linear relation between absolute density estimates provided by the quadrat sampling and the time-based relative abundance counts.

### Comparison of time-based and quadrat sampling

Model ranking using the AICc shows strong support for saturating-type transfer functions between time-based relative density estimates and quadrat absolute density averages for all snail species and habitats (Table 3 and S1 Table). We also performed the statistical analysis of the combined *Bulinus* spp. and *B. pfeifferi* counts for the stream in Panamasso. The weakest support for saturating-type functions was found for *Bulinus* spp. in the stream (probability 0.69), mainly driven by the very high quadrat densities recorded in March 2015 (Fig 3). Interestingly, when considering counts of *Bulinus* spp. and *Biomphalaria pfeifferi* together presented larger support for a saturating effect rather than a linear trend (probability 0.94). Support for saturating vs. non-saturating models was high (> 0.75 probability in all species-habitat configurations) even when considering only data in the range of low time-based counts where visual inspection could suggest adequacy with linear-type functions (S3 Fig). The unconditional estimates of the plateau value of the saturation functions for the quadrat-based absolute densities are given in S2 Table. The value of the plateau for *Bulinus* spp. in the river were strongly uncertain with large confidence intervals, reflecting the fact that the observed densities did not cover a range over which the saturation was visible (Fig 4). We stress the fact that these do not correspond directly to observed absolute densities since they do not take into account zero-inflation. In fact, in terms of measurement models, the zero-inflated family (Poisson and Negative Binomials) consistently had the strongest support for all species and habitats (Table 3). For *Bulinus* spp. in the ephemeral habitats as well as the combined counts in the stream the strongest support was for a constant zero-inflation parameter *σ*. The unconditional estimates of *σ* were of the order of 20%-40% depending on snail species and habitat and with varying degrees of uncertainty (S3 Table). Estimates of *σ* were less uncertain for *Bulinus* spp. in the temporary pond (95% confidence intervals, CI 0.28-0.49). On the other hand there was strong support for a varying zero-inflation probability for *B. pfeifferi* and *Bulinus* spp. in the permanent stream. We highlight the fact that the confidence intervals were computed based on the unconditional parameter variance which accounts for model uncertainty [61].

**Table 3:** Model structure probabilities. The probability that each model lies within the 95% confidence set were computed by summing AIC weights for the functional form of the transfer function and for zero-inflation in the measurement model. The probability of constant vs. logistic zero-inflation parameter was computed using the ratio of their respective AIC weights to their sum.

Species	Habitat	Functional form	Measurement model	
		Saturation	Zero-Inflation	Constant vs. Logistic
*Bulinus*	Pond	1.00	0.99	0.71
*Bulinus*	River	0.83	0.89	0.74
*Biomphalaria*	Stream	0.92	0.93	0.56
*Bulinus*	Stream	0.69	0.55	0.35
both	Stream	0.94	0.58	0.61

**Fig 5:**
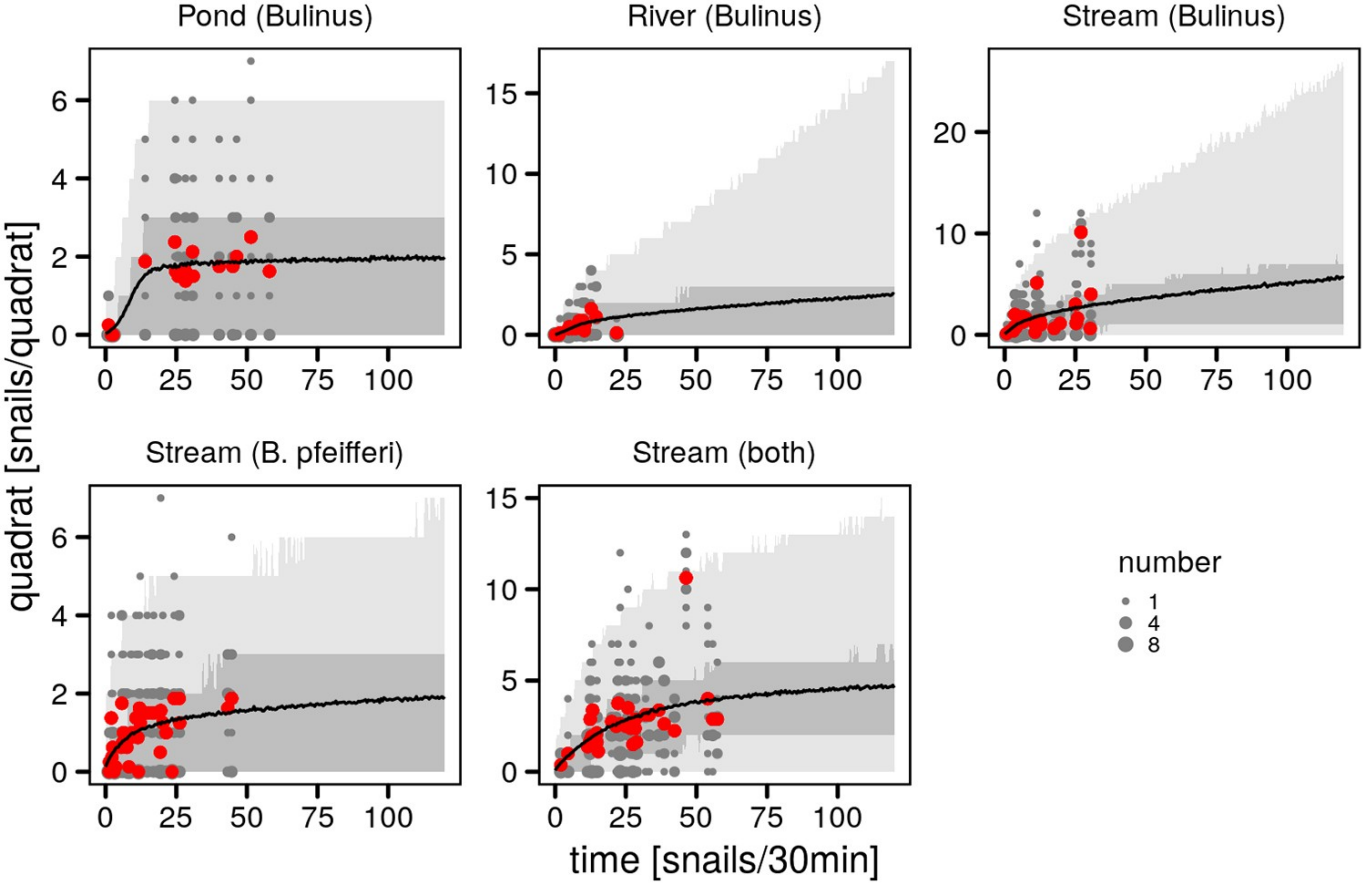
Model simulations of the relations between time-based and quadrat sampling strategies. Simulations were performed using the 95% credible model set (S1 Table) for each species-habitat configuration. Simulations are shown in terms of the mean (black line) as well as the 95% (light gray ribbon) and 50% (dark gray ribbon) simulation envelops for 5000 simulations per time-based count. Quadrat counts (gray dots) are given as in the first row of Fig 3 along with monthly means (red dots).

Simulations confirmed the adequacy of the inferred link between time-based and quadrat counts (Fig 5). Overall the tendency of the mean quadrat count saturation is visible for all species and habitats. The effect of the zero-inflation measurement model is illustrated by the inclusion of 0 within the 50% simulation envelop across values of time-based counts except for the *Bulinus* spp. and the combined counts in the stream for which zero-inflation had much less support (both with probability 0.58). The uncertainty in the saturation value of quadrat counts for *Bulinus* spp. in the river is illustrated here by large 95% ensemble simulation envelops. Ensemble simulations therefore support the statistical link between time-based and quadrat counts, in particular with respect to the saturation of quadrat-based counts. The relationship between the time-based counts and the estimated mean absolute density is given in S4 Fig.

## Discussion

In this work we re-introduce the use of a quadrat-based sampling protocol for the estimation of absolute abundance of the intermediate snail hosts of schistosomiasis across different climatic zones and habitat types. Contrasting the results from this method with more widely-used time-based sampling strategies allowed for the evaluation of the agreement between these two methods, in particular with respect to the quantification of seasonal variations in snail abundance as well as the detection of density feedbacks.

Results show that time-based and quadrat sampling techniques yield coherent signals of snail abundance seasonality across habitats and species. The 3.5-year dataset presented here confirms the asynchronous pattern between the permanent and ephemeral habitats we have previously reported for the 2014-2015 window [24]. Indeed *Bulinus* spp. in temporary habitats peak during the rainy season in Burkina Faso (July-October) with populations collapsing at the end of the year with the progressive shrinkage of their aquatic habitat. On the other hand *Bulinus* spp. and *Biomphalaria pfeifferi* in the permanent habitat in the South-West tend to peak during the warm dry season in March-April. For *Bulinus* spp. in the permanent stream a post-rainy season peak was also visible both in the quadrat and time-based counts which had not appeared as clearly in the 2014-2015 dataset. Although both sampling methodologies yielded similar results, the time-based counts tended to have larger fluctuation amplitudes with respect to the quadrat-based density estimate, as highlighted by the correlation analysis.

The statistical analysis of the relationship between time-based and quadrat counts provided strong support for the hypothesis that quadrat-based estimates of absolute snail density have a saturating behavior with respect to time-based counts. Both the measured and simulated quadrat-based estimates of the mean absolute densities are consistent with other estimates of absolute snail density available in the literature, with values in the range of 25-75 snails/m^2^ (Table 1). More interestingly, the saturating behavior of the estimated absolute densities provides strong support for the importance of density feedbacks in natural snail populations that have been a matter of controversy in the literature [22]. Our previous analysis of the timeseries of time-based counts had already detected negative density feedbacks using ecological model selection for both snail genera across natural and man-made habitats [24].

Here the saturation observed in quadrat-based density estimates provides additional evidence for the importance of habitat-dependent carrying capacity on snail population dynamics in both permanent and ephemeral aquatic environments. In fact, although the specific values of the density plateaus of our statistical analysis present large confidence intervals (S2 Table), there exists strong support of saturation for all species and habitats except for *Bulinuns* spp. in the permanent stream. An important question beyond the scope of this study is whether *Bulinus* spp. and *Biomphalaria* spp. co-occurrence in permanent habitats such as the stream in Panamasso entails competition dynamics between the two genera, as suggested by the support for saturating functional forms for the combined snail counts.

The question remains on the underlying causes of the additional variability in the output of the time-based sampling protocol. This additional variability could be due to systematic variations in the factors that influence the ease with which habitats can be sampled (vegetation, water turbidity, water depth), or on snail identifiability (size) which do not reflect actual variations in absolute densities. The statistical analysis yielded strong support for zero-inflated quadrat counts with a constant zero-inflation parameter in the ephemeral habitats (pond and river) and a varying parameter value in the permanent stream. Our main hypothesis for the presence of zero-inflation in quadrat counts is that the clustered distribution of snails entails a certain probability of detection of a cluster, with the non-detection of a cluster accounting for the additional 0 counts. Other processes could yield zero-inflation in counts, for instance due to limits in the detectability of juvenile snails by scooping. Additional experiments would be necessary to elucidate this point, in particular the quantification of snail aggregation across habitat types in other settings.

If the hypothesis of snail clustering is retained, it does not support a systematically varying density of snail clusters with local carrying capacities. As an alternative hypothesis, the variation of snail sizes could explain the systematic variations in time-based densities. Indeed, in the ephemeral habitats time-based densities at the beginning of the rainy season are typically larger than in the following months [29]. This could reflect the fact that the snail populations that first colonize the habitat are the aestivating adults from the previous year, which are then replaced by a new generation of young snails in the following months [29]. A similar argument could be made for the permanent habitat with the alternation of growing and egg-laying periods of the year as a function of temperature [22]. This hypothesis would need to be verified by the analysis of snail sizes collected by both sampling strategies. The result of this analysis therefore suggests that the time-based protocol used in this study is biased towards the detection of larger adult snails and would justify the improvement of the method with more systematic searches, as recently proposed elsewhere [62].

A limitation of the quadrat-based sampling strategy was that the obtained precision on the mean absolute densities (relative SE ≈40%) was much lower than the one computed *a priori* for the determination of the number of quadrat replicates (8) to make at each sampling date (relative SE of 20%). This was mainly due to the over-estimation of snail densities based on the data from the literature (≈15 vs. 67 snails/m^2^), despite a lower value of the index of dispersion (≈1.5 vs. 2). This mismatch stresses the challenge of the design of quadrat-based sampling based on *a priori* estimates of absolute density as well as snail clustering in space. The analysis of detailed snail location data such as the one proposed in the Supplementary Material (S1 Appendix) would have been valuable in the design phase of the sampling protocol. However in this study the analysis was only done retrospectively. We would therefore recommend that similar analysis be done prior to the design of future protocols.

The major limitation to the implementation of *a priori* analysis of snail clustering patterns is the availability of the type of habitat-scale data on snail location as the one in [41]. In the absence of this type of data, we would recommend to do preliminary sampling to have approximate estimates of both the mean snail density and the index of dispersion for a chosen quadrat size. Data from the latter study made in Tanzania may not adequately approximate the climatic and hydrological conditions of our study sites in Burkina Faso. The pluviometry of the Southwestern site of Panamasso is comparable to that of Tanzania, with the presence in both sites of *Biomphalaria pfeifferi*. However the more ephemeral conditions in the Eastern site of Lioulgou are clearly distinct from the Tanzanian site. This stresses for the need of the continued investment in malacological research and the collection of data across habitat types in schistosomiasis endemic countries.

Another element that was not considered in this study is the effect of substrate variations in the design of the quadrat sampling protocol. Indeed *B. pfeifferi* has been shown to prefer solid supports (such as wood and leaves) and bedrock to sand and gravel [41]. Moreover there are accounts of the preference of the intermediate hosts for plastic waste [63], although no quantification of this preference exists to our knowledge. In this perspective a limitation of this study was to not have done specific trials to determine the degree of randomness obtained using our procedure for the random positioning of sampling quadrats, in particular the effect of the repositioning of the quadrat if it didn’t land within the predefined distance to the shoreline with respect to the snail species’ substratum preferences. The values of the index of dispersion observed in the habitats sampled in this study were not particularly high except for *Bulinus* spp. in the temporary pond, which do not indicate strong spatial heterogeneities. However the effect of variations of substratum type and in particular that of natural and plastic supports could justify the use of stratified sampling schemes in other contexts should snail densities per substratum vary significantly. We would therefore recommend for the design of future protocols to include a procedure for the testing of the quadrat positioning method with respect to the snail species’ and habitat characteristics, including water depth and substratum preferences. We hope that the methodological considerations provided in this work as well as the results will serve as support for the implementation of similar sampling strategies in other data-constrained settings.

Taken together the results of this work provide several take home points from the schistosomiasis control perspective. For the specific context of Burkina Faso, the confirmation by both methods of strong seasonal variations of snail populations with different peak timings calls for additional investigations into the timing of chemotherapy depending on climatic zone and habitat type. In temporary habitats the absence of snails during the dry season offers a clear window for treatment. On the other hand in permanent habitats in the Southwestern parts of the country [50], transmission minima should be identifyed using snail shedding or PCR techniques [33]. In fact snail abundance per se does not determine transmission risk, but rather the density of infected snails which we did not measure in this study. However a first approximation of the time of minimum transmission could be 1-2 months (length of the prepatent period) after minimal snail densities, i.e. approximately in September-October in the case of Panamasso. This should however be verified using appropriate snail infection sampling techniques.

In the perspective of implementing snail control measures different scenarios emerge in the different habitats. The most problematic is the temporary pond since snail densities seem to be at carrying capacity for most of the period when snails are present which calls for a thorough control protocol due to the potential for population rebounds. On the other hand *Bulinus* spp. densities did not appear to be subject to the same strength of density feedbacks. Finally in the permanent stream both snail genera present seem to be subject to density feedbacks at least during part of the year, in particular for snail densities above approximately 10 snails/m^2^ for *B. pfeifferi* and 20 snails/m^2^ for *Bulinus* spp. which typically occurred between October and March. Beyond the problem of compensatory feedbacks, the observation of density feedbacks warrants the evaluation of the effect of reductions in snail populations in terms of transmission potential to humans as subject to non-linearities in cercarial productivity with resource availability [35, 36]. Novel approaches leveraging DNA-based tools such as PCR for the identification of snail and schistosome species [3], as well as environmental-DNA (eDNA) quantification techniques recently developed for schistosomes can play in important role in investigating these issues [64].

Finally, the comparative analysis of time-based and quadrat protocols provides a basis for the choice of snail sampling strategy for the schistosomiasis research and control programs in settings with seasonal climates such as the one of Burkina Faso. Regarding the detection of seasonal variations the methods were in large agreement in particular for the temporary pond and the ephemeral stream. On the other hand the outputs of the two methods were less correlated in the permanent stream in particular at low time-based counts. This pattern could be linked to the larger support for varying zero-inflation probability in the permanent habitat with respect to constant zero-inflation probability in the temporary habitats. Assuming zero-inflation is produced by snail spatial clustering as argued above, this potentially suggests more complex ecological dynamics in the permanent habitat with varying snail aggregation patterns during the year which in turn have an impact on the outputs of time-based sampling. One possible explanation could be the overall stronger hydrological variability in the small stream with respect to the larger ephemeral river.

Subject to the above-mentioned limitations of this study, the following takeaways regarding the choice of snail sampling protocols can be drawn:

- For the purpose of monitoring seasonal populating variations classic time-based sampling provide adequate relative abundance estimates in hydrogically ephemeral environments which present clear favourable and unfavorable conditions such as temporary ponds and ephemeral rivers. Time-based sampling can therefore be preferred to the more cumbersome quadrat sampling technique in these contexts.
- Quadrat sampling provides more accurate estimates of snail abundance seasonality in small permanent streams which present strong hydrological variations. Time-based sampling are not recommended in this type of habitat because they appear to be affected by variations in snail clustering which offsets the correspondence with absolute density estimates.
- In the perspective of the implementation of snail control measures, quadrat sampling provides the additional benefit of the detection of density feedbacks in snail population dynamics as well as comparable measures of intervention efficacy between sites.
- The design of quadrat sampling protocols should take into consideration potential variations in snail clustering and its effect on the precision of mean absolute density estimates as a function of the number of quadrat replicates.

Traditional protocols of ecological monitoring for the population of the snail intermediate hosts of human schistosomes will continue to be the backbone of control and elimination trials in endemic countries. Through this study we re-propose the quadrat-based sampling method which is well-suited for shallow water habitats such as temporary ponds and rivers and streams with gentle slopes. The agreement between the quadrat-based and time-based density estimates in terms of the seasonal variations of snail densities is encouraging in its use for the quantification of disease transmission windows. Importantly quadrat sampling allowed for estimates of the absolute snail density which were comparable across field sites, and highlighted a saturation in absolute densities at high time-based snail counts. Traditional ecological sampling methods will be a centerpiece of schistosomiasis control programs aiming at transmission interruption. The results of this work suggest that the systematic comparison of alternative sampling protocols is an important step in supporting this aim.

## Supporting information

S1 Appendix Re-analysis of spatial presence data of *Biomphalaria pfeifferi* in [41]. (PDF)

S1 Fig Seasonality of population dynamics. Data are presented by intermediate host genera for the quadrat sampling (top row, snails/30x30cm quadrat) and time-based method (bottom row, snails/30min search) along with the coefficient of variation (CV) of the monthly count means with the indicative timing of the rainy season (July-September) (blue rectangles).
(TIF)

S2 Fig Correlation between monthly mean densities from each sampling technique. Pearson (top row) and Spearman (bottom row) correlations computed for increasing ranges of time-based mean counts.
(TIF)

S3 Fig Support for saturation in quadrat counts for different ranges of time-based counts. Support was computed as in S2 Table. Each point corresponds to the support of saturating vs. non-saturating models when considering only sampling data within the range of time-based counts. The absence of points bellow 20 snails/30min are due to the absence of data.
(TIF)

S4 Fig Model simulations of the relations between time-based and absolute snail densities estimated by quadrat sampling. Simulations were performed using the 95% credible model set (S1 Table) for each species-habitat configuration. Simulations are shown in terms of the mean (lines) for 5000 simulations per time-based count.
(TIF)

S1 Table Model selection results. (PDF)

S2 Table Estimates of the saturation of the transfer function between time and quadrat counts. Unconditional mean and 95% confidence intervals (in parenthesis). Note that these values do not correspond to absolute densities which require in addition the accounting for zero-inflation in counts.
(PDF)

S3 Table Zero-inflation parameter estimates. Unconditional mean and 95% confidence intervals (in parenthesis).
(PDF)
