# Supplementary material for: Comparative analysis of time-based and quadrat sampling in seasonal population dynamics of intermediate hosts of human schistosomes
Source: PLoS Negl Trop Dis. 2019 Dec 20;13(12):e0007938. doi: 10.1371/journal.pntd.0007938 (PMC6957212; doi:10.1371/journal.pntd.0007938)
Supplement: S1 Appendix — (PDF) [file pntd.0007938.s001.pdf]

Supplementary Material
Comparative analysis of time-based and quadrat sampling in seasonal population dynamics of intermediate hosts of human schistosomes

Javier Perez-Saez, Theophile Mande, Dramane Zongo, Andrea Rinaldo

1 Spatial point process analysis of snail occurrence

We here provide support for the choice of quadrat sampling scheme described in the main text through the analysis of detailed data on the spatial occurrence of snail intermediate hosts individuals from the literature. We aim at assessing quantitatively the existence and scale of aggregation of snails in natural habitats which has been studied at river stretch-level by (Woolhouse and Chandiwana, 1989) (index of dispersion in the range 1.8-8), but for which no quantitative analysis at the micro-habitat level exists in the literature to our knowledge.

1.1 Data

The analysis is based on data reported by Utzinger et al. in (Utzinger and Tanner, 2000) regarding the study of the micro-habitat preferences of *Biomphalaria pfeifferi* and *Lymnaea natalensi* in natural and man-made habitats in Tanzania. The sampling protocol they used consisted of an exhaustive sampling of the 11.7m^2 of a natural habitat, an isolated pool of a river, and the recording of the position of each individual snail relative to the left shore of the habitat and the distance in the x-direction, and distance along the main axis of the habitat from the first sampling transect in the y-direction, as well as the width of the habitat at each value of y (SM Figure 1). The position in the x-direction needed to be converted to a an absolute value instead of the distance to the left shore since the latter cannot be assumed to be a straight line. As a working hypothesis we assumed that the habitat is symmetric along its main axis, which permitted the delineation of the left shore as half the reported width of the habitat in the x-direction. The location of individual snail occurrence could then be plotted in a reference system with origin the center of the bottom edge of the habitat (SM Figure 1).

1.2 Spatial point process analysis

In this study we were interested in the probability density of sampling an individual snail accounting both for location-dependent factors to test the existence and extent of spatial clustering of individuals. The analysis of the presence data of *B. pfeifferi* was framed in terms of *point process* models (PPMs), meaning that each record of snail location is considered as a point event, where the number and location of points are random (Baddeley et al., 2015). The intensity $\lambda(s)$ of the process at location s within a study area $\mathcal{A}$ is a measure of reported abundance which is proportional to the probability density $f(s)$ of an event occurring at that location relative to all other locations in $\mathcal{A}$ ($f(s) = \lambda(s)/\int_{\mathcal{A}} \lambda(s)ds$). The simplest PPM is a Poisson model with constant intensity, which corresponds to the complete spatial randomness (CSR) assumption often used as null hypothesis in PPM testing.

Covariates The intensity can be made location-dependent to incorporate the effect of the characteristics of the habitat on snail abundance. In fact Utzinger and Tanner (2000) showed strong heterogeneity in *B. pfeifferi* in the habitat with individuals nearly exclusively sampled in water depths shallower than 10cm

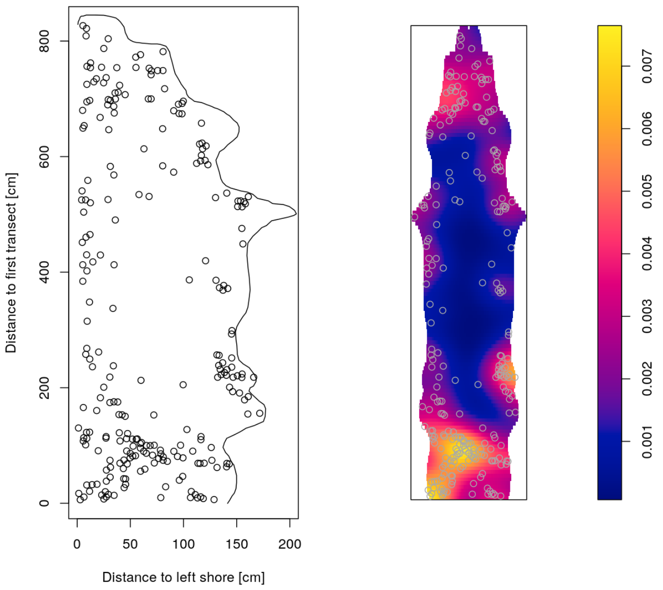

SM Figure 1: Spatial point data of occurrence of *Biomphalaria pfeifferi*. The data consists of point occurrence data of individual snails in an isolated pool in Tanzania reported in (Utzinger and Tanner, 2000). (*Left*) Raw digitized data from (Utzinger and Tanner, 2000). (*Right*) Plot of data in absolute coordinates (points) and density of points per cm^2 (colorcode) assuming the habitat was symmetric along its main axis (cf. section 1.1)

with a decreasing density as depth increased. A similar trend was detected for distance to river shore although with a weaker slope. In the absence of data of water depth data we here considered three sets of covariates to account for spatial heterogeneity in intensity distance to river shore, and distance to the center of the habitat, and relative distance to the center. The relative distance to the center corresponds to the distance in terms of the fraction of distances in the x and y directions divided by the maximum distances in each direction. It can be seen as a proxy of water depth assuming that the deepest point of the habitat is in its center and constant slopes from the center to the edges. We here tested both linear and cubic relations between the covariates and the log-intensity, as well as the linear combination of distances to shore and to center and their cubic transformation.

Models of clustering The most commonly used family of models that consider clustered point processes are the Neyman-Scott processes (Neyman and Scott, 1958). They assume that the observed events originate from a parent unobservable Poisson process which generates cluster seeds, around each of which daughter events occur according to a given probability density function of distance to the cluster's seed. We here test three different cluster process (see (Baddeley et al., 2015) for detailed descriptions). Matérn's process assumes that daughter events occur independently and uniformly within a disc of radius r around the cluster seed. The Thomas (or Gaussian type) process assumes that daughter events occur around parents points following a bivariate Gaussian distribution with zero mean and covariance matrix $I_{2\times2}$, where $\sigma I_{2\times2}$ is the 2x2 identity matrix. Finally the Cauchy process assumes that daughter points occur following the 2-dimensional Cauchy distribution with location 0 and scale parameter σ. The Cauchy distribution is characterized by fatter tails than the Gaussian distribution, i.e. presenting more frequent events at large distances from the parent point. In all three cluster processes it is therefore possible to evaluate the characteristic scale of clustering, as well as the expected number of events per cluster. We fit all combinations of cluster models as well as the Poisson process with covariates and their transformations

using the `spatstat` package in R by optimizing the Palm likelihood (Tanaka et al., 2008). Given that this method aims at Maximum Likelihood estimates, we then compare models using the AIC criterion following (Tanaka et al., 2008), which reads:$AIC = -2 \times \log(\mathcal{L}_{Palm}) + 2 \times k$, where $\mathcal{L}_{Palm}$ is the Palm likelihood, and k the number of parameters of the model. The models are then ranked in descending AIC order, lower values of AIC corresponding to larger support. Relative support for each model can then be computed with the AIC weights $w_i = \frac{e^{-0.5\Delta\mathrm{AIC}_i}}{\sum_j e^{-0.5\Delta\mathrm{AIC}_j}}$, where ΔAIC denotes the difference between the the AIC of model i and the minimum AIC score among all tested models (Burnham et al., 2011).

Measures of clustering and goodness of fit A widely used measure of clustering for PPMs is Ripley's K function which consists of an estimate of the second-order moment of the process, i.e. the variance of the inter-point distances. The K-function is linked to the conditional probability of observing additional events knowing that an event occurred at location s within a radius r. In the case of a homogeneous Poisson process this is given by $K(r) = \lambda^{-1}\mathbb{E}[\text{number of events within r}]$. Since the expected number of events within a disc or radius r is πr^2 in the case of CSR, values of $K(r) > \pi r^2$ indicate the presence of clustering at distance r. If the intensity varies in space an inhomogeneous generalization of the K-function can also be estimated (Baddeley et al., 2015). We here use a variance-stabilized transformation of the K-function, the L-function which reads $L(r) = \sqrt{K/\pi}$, who's expectation at distance r is equal to r. Values of $L(r) - r > 0$ indicate clustering at radius r.

The departure of the observed L-function and the one resulting from simulations of the fitted PPM can be used to evaluate model goodness-of-fit by building significance tests based on simulation envelops (Baddeley et al., 2014). We here employ the Diggle-Cressie-Loosmore-Ford (DCLF) statistic based on the sum of squared differences between the observed and modelled L-functions to build p-values for the fitted models (see (Baddeley et al., 2014) for details). In this framework the p-value corresponds to the null hypothesis that the data comes from the simulated model, therefore p-values < 0.05 indicate that the model should not be retained. Confidence intervals for the parameters of the Neyman-Scott models specifying cluster intensity and size were estimated by parametric boostrap with 1000 repetitions as in (Yue and Loh, 2015).

2 Results

2.1 Model selection

Three models stand out in terms of Akaike weights from the list of 36 tested models. All three are Neyman-Scott cluster type models with the Cauchy and the Thomas models dominating the model set, with probabilities 0.44 and 0.39 respectively of being the best model for the data (in terms of Kullback-Leibler divergence, see (Burnham et al., 2011), SM Table 1). Model selection through the AIC indicated very clear support for the inclusion of relative distance to the shore with respect to the other covariates, with all other parameter models having negligible Akaike weights. All three models incorporate relative distance to the shore through a cubic polynomial, with intensity sharpely increasing for distance values up to 0.8 and then a slight decrease for values larger than 1 (SM Figure 2). The resulting spatially-varying intensity map is given in SM Figure 2.

2.2 Model clustering

Neither the Cauchy (p-value = 0.415) nor the Thomas (p-value = 0.330) were rejected by the DCLF test, thus supporting their goodness of fit with respect to the clustering patterns in the data. For comparison the inhomogeneous Poisson model was rejected by the DCLF test (p-value = 0.03). visual inspection of the L-function for these three models confirms the result of the test, with the 95% simulation envelopes of the inhomogenous Poisson model failing to cover the clustering of *B. pfeifferi* between 10cm and 20cm distances as well as at 40cm (SM Figure 3). Moreover the mean of the simulations predicts a slight

SM Table 1: Model fit results. Results given in terms of the Akaike Information Criterion (AIC), the difference of AIC with the best-performing model ($\Delta_i = AIC_i - minAIC$), and the AIC weight ($w$).

	Model	Covariate	Trend	AIC	Δ	w
1	Cauchy	reldist_to_center	cubic	23696.89	0.00	0.44
2	Thomas	reldist_to_center	cubic	23697.11	0.23	0.39
3	MatClust	reldist_to_center	cubic	23698.72	1.83	0.17
4	Cauchy	reldist_to_center	linear	23937.05	240.16	0.00
5	Thomas	reldist_to_center	linear	23937.57	240.69	0.00
6	MatClust	reldist_to_center	linear	23939.03	242.15	0.00
7	Cauchy	dist_to_center	cubic	23952.56	255.67	0.00
8	Thomas	dist_to_center	cubic	23952.68	255.80	0.00
9	MatClust	dist_to_center	cubic	23954.33	257.45	0.00
10	Cauchy	dist_to_center	linear	23968.09	271.21	0.00
11	Thomas	dist_to_center	linear	23968.40	271.51	0.00
12	MatClust	dist_to_center	linear	23969.94	273.06	0.00
13	Cauchy	comb	bilinear	24009.66	312.77	0.00
14	Thomas	comb	bilinear	24010.31	313.42	0.00
15	MatClust	comb	bilinear	24011.69	314.81	0.00
16	Cauchy	comb	bicubic	24034.15	337.26	0.00
17	Thomas	comb	bicubic	24034.37	337.48	0.00
18	MatClust	comb	bicubic	24035.97	339.09	0.00
19	poisson	reldist_to_center	cubic	24101.13	404.24	0.00
20	Cauchy	constant	homogeneous	24113.85	416.96	0.00
21	Thomas	constant	homogeneous	24115.27	418.39	0.00
22	MatClust	constant	homogeneous	24115.88	418.99	0.00
23	Cauchy	dist_to_shore	linear	24298.82	601.94	0.00
24	Thomas	dist_to_shore	linear	24300.22	603.33	0.00
25	MatClust	dist_to_shore	linear	24300.88	603.99	0.00
26	Cauchy	dist_to_shore	cubic	24333.70	636.81	0.00
27	Thomas	dist_to_shore	cubic	24335.04	638.15	0.00
28	MatClust	dist_to_shore	cubic	24335.79	638.90	0.00
29	poisson	dist_to_center	cubic	24341.96	645.07	0.00
30	poisson	dist_to_center	linear	24391.56	694.68	0.00
31	poisson	reldist_to_center	linear	24393.30	696.42	0.00
32	poisson	comb	bicubic	24437.52	740.63	0.00
33	poisson	comb	bilinear	24492.49	795.61	0.00
34	poisson	constant	homogeneous	25017.40	1320.52	0.00
35	poisson	dist_to_shore	cubic	25134.54	1437.65	0.00
36	poisson	dist_to_shore	linear	25160.13	1463.24	0.00

regularity in the PPM at odds with the observed pattern for distances larger than 3cm. On the other hand both the Cauchy and Thomas cluster processes successfully encompass the observe L values with the mean of the processes following the increasing values of L from 0 to around 20cm. The main difference between the two models is the decrease in L values for the Thomas model at large values of $r(> 35cm)$ which does not happen in the Cauchy process, the latter better matching the observed trend in L which plateaus at $r > 45cm$. This effect is possibly due to the fatter tails of the Cauchy distribution. The estimated scale parameters of the cluster sizes where of 8.84cm (CI 5.81-12.1) for the Cauchy and of 9.34cm (CI 6.98-11.6) for the Thomas processes. As shown by cumulative distribution functions of distance of daughter events to cluster center of each model, the Thomas process predicts much more concentrated cluster events (SM Figure 4).

2.3 Simulation of quadrat sampling protocols

In the perspective of evaluating quadrat sampling protocols, we here use the Cauchy Neyman-Scott process to simulate the spatial occurrence of snail, on which we simulate a random quadrat sampling protocol. We test quadrat sizes from 15x15cm to 40x40cm, as well as 2 to 20 replicas per sampling. We the record the mean snail density, the standard deviation and the index of dispersion both for the simulated point process as covered by a grid of cell size given by the quadrat size, here considered as the reference, and the same statistics estimated by the random quadrat sampling protocols on each realization of the point process. We perform 100 simulations of the point processes and 100 random placements of the grid,

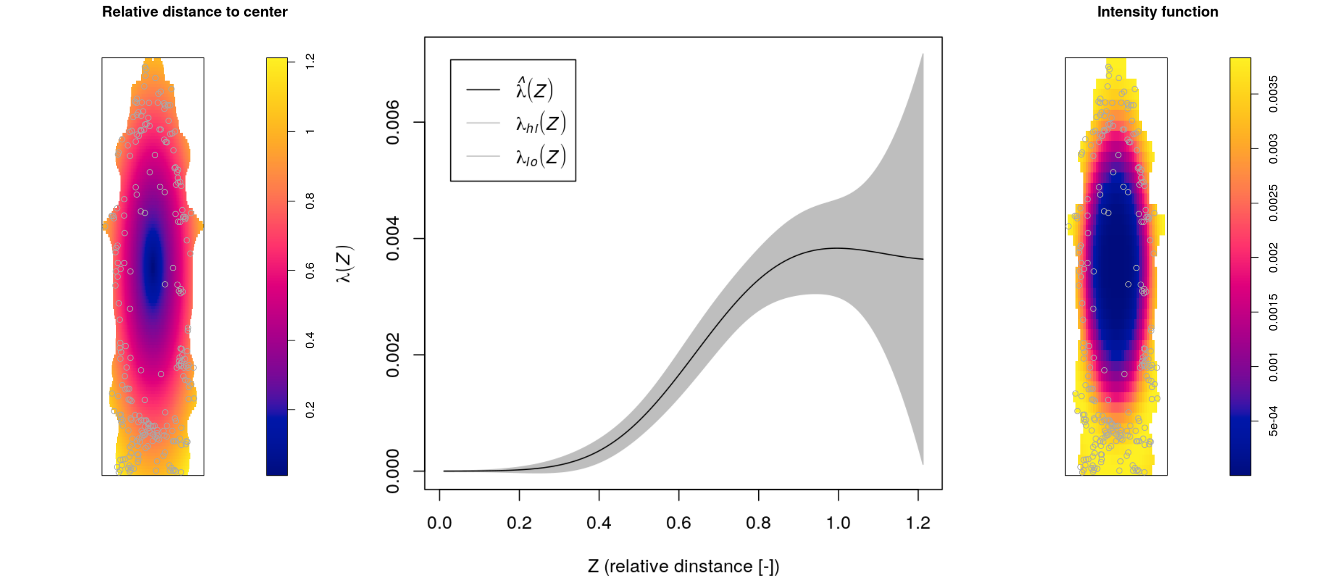

SM Figure 2: Inhomogeneous intensity process. (*Left*) Map of relative distance to center consistently selected among candidate covariates. (*Middle*) Fitted cubic polynomial of intensity as a function of relative distance (line) along with 95% prediction interval (shading). (*Right*) Predicted map of spatilly-varying intensity using the mean of the intensity function.

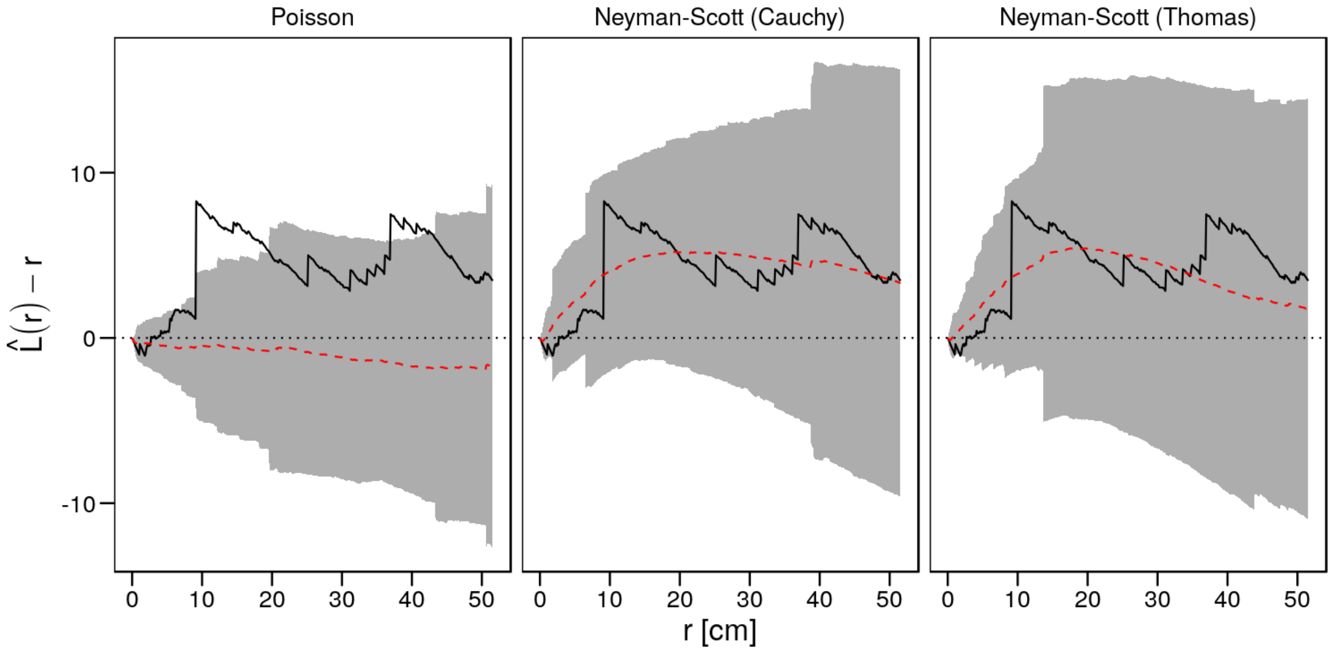

SM Figure 3: L-function plots. The L-functions were estimated for the inhomogeneous intensity for the observed events (solid black line), and assuming a Poisson process for the events (*Left*), and for the two best-porfming cluster models (*Middle* & *Right*). Simulation envelops of the L-function correspond to the 95% inter-quantile range (gray shading) of 199 realisations, along with the mean of the simulations (dotted red line).

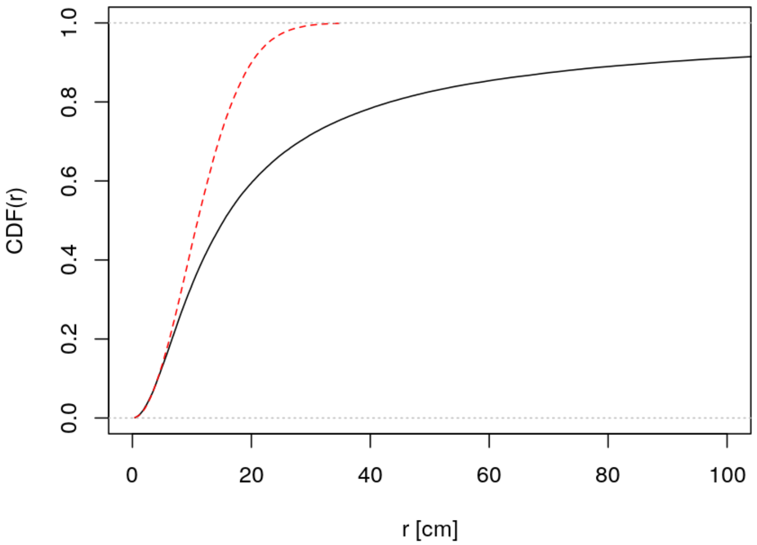

SM Figure 4: Cumulative distribution function of the distance of daughter events to the cluster center for the fitted Cauchy (solid black line) and Thomas (dotted red line) processes.

and 100 simulations of the quadrat sampling for each point process simulation and each grid placement (results in SM Figure 5).

The standard error on the mean of the simulated quadrat sampling scheme decreases with the number of quadrat replicas, however this decrease is much stronger for low number of replicas, and then stagnates for more than 15 replicas as predicted by the theory (see main text). The standard error is systematically lower for quadrat sizes larger than 25x25cm.

3 Discussion

Using detailed records of snail occurrence in space and PPM we have shown that *B. pfeifferi* presents a clustered distribution in natural habitats. This result has practical implications for the design of sampling protocols in the perspective of monitoring schistosomiasis control trial outcomes.

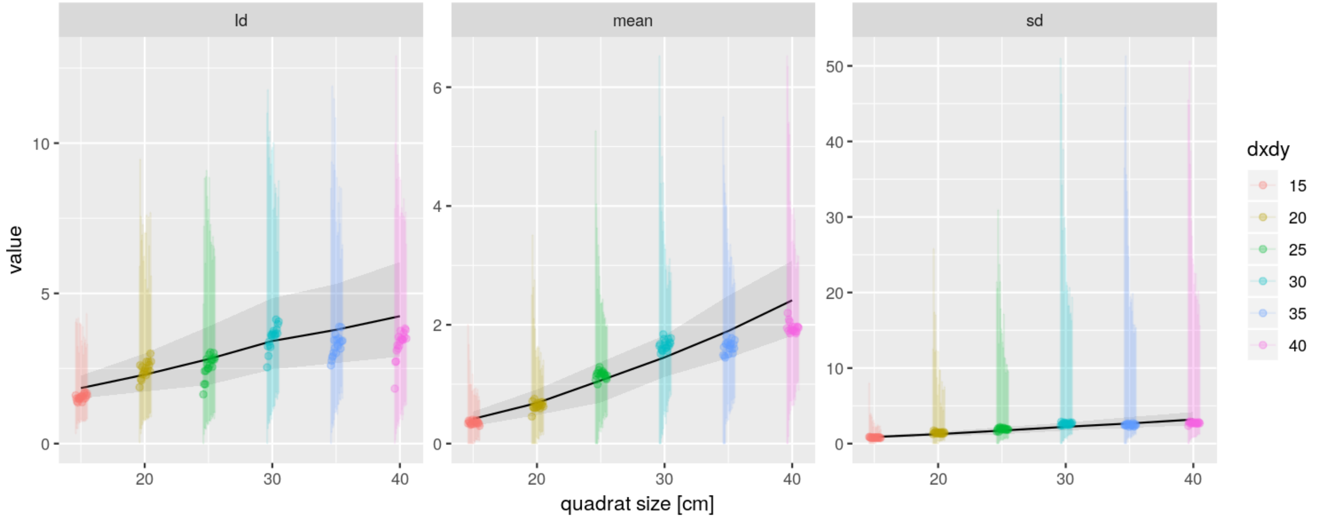

SM Figure 5: Results of the simulations of quadrat sampling in terms of mean (snails/quadrat), index of dispersion (Id), and standard deviation (sd). The statistics are estimated both over a grid (line, shading gives the 95% bootstrap CI), and a random quadrat sampling protocol with different number of replicas (dots, error bars give the 95% bootsrap CI), from 2 to 20.

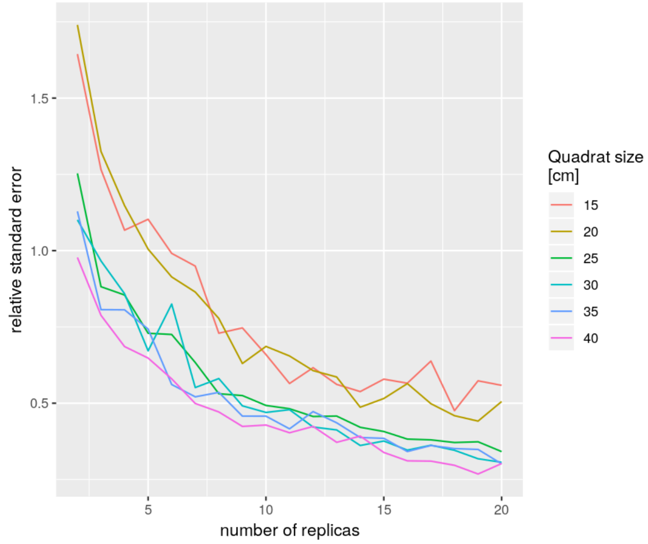

SM Figure 6: Simulated relative standard error of quadrat sampling schemes

References

A. Baddeley, P. J. Diggle, A. Hardegen, T. Lawrence, R. K. Milne, and G. Nair. On tests of spatial pattern based on simulation envelopes. *Ecological Monographs*, 84(3):477–489, 2014. doi: 10.1890/13-2042.1.

A. Baddeley, E. Rubak, and R. Turner. *Spatial Point Patterns: Methodology and Applications with R*. Chapman and Hall/CRC Press, London, 2015.

K. P. Burnham, D. R. Anderson, and K. P. Huyvaert. AIC model selection and multimodel inference in behavioral ecology: some background, observations, and comparisons. *Behavioral ecology and sociobiology*, 65(1):23–35, 2011. doi: 10.1007/s00265-010-1084-z.

J. Neyman and E. L. Scott. Statistical approach to problems of cosmology. *Journal of the Royal Statistical Society: Series B (Methodological)*, 20(1):1–29, 1958. doi: 10.1111/j.2517-6161.1958.tb00272.x.

U. Tanaka, Y. Ogata, and D. Stoyan. Parameter estimation and model selection for Neyman-Scott point processes. *Biometrical Journal: Journal of Mathematical Methods in Biosciences*, 50(1):43–57, 2008. doi: 10.1002/bimj.200610339.

J. Utzinger and M. Tanner. Microhabitat preferences of Biomphalaria pfeifferi and Lymnaea natalensis in a natural and a man-made habitat in Southeastern Tanzania. *Memórias do Instituto Oswaldo Cruz*, 95(3):287–294, jun 2000. ISSN 0074-0276. doi: 10.1590/S0074-02762000000300002.

M. E. J. Woolhouse and S. K. Chandiwana. Spatial and temporal heterogeneity in the population dynamics of *Bulinus globosus* and *Biomphalaria pfeifferi* and in the epidemiology of their infection with schistosomes. *Parasitology*, 98(01):21–34, 1989. doi: 10.1017/S003118200005965.

Y. Yue and J. M. Loh. Variable selection for inhomogeneous spatial point process models. *Canadian Journal of Statistics*, 43(2):288–305, 2015. doi: 10.1002/cjs.11244.
