# Supplementary figures and images for: Comparative analysis of time-based and quadrat sampling in seasonal population dynamics of intermediate hosts of human schistosomes
Source: PLoS Negl Trop Dis. 2019 Dec 20;13(12):e0007938. doi: 10.1371/journal.pntd.0007938 (PMC6957212; doi:10.1371/journal.pntd.0007938)

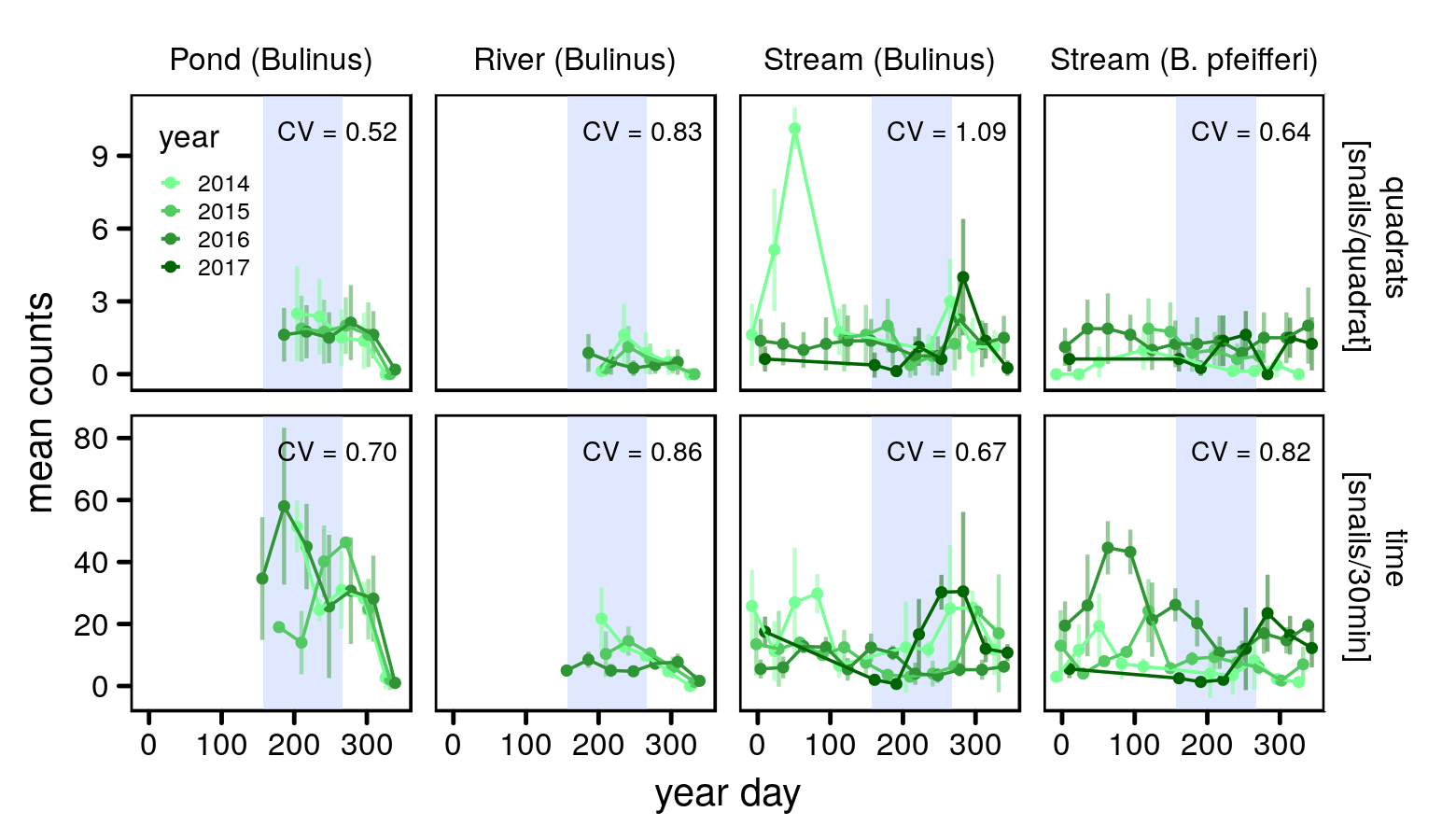

Supplement: S1 Fig — Data are presented by intermediate host genera for the quadrat sampling (top row, snails/30x30cm quadrat) and time-based method (bottom row, snails/30min search) along with the coefficient of variation (CV) of the monthly count means with the indicative timing of the rainy season (July-September) (blue rectangles). (TIF) [file pntd.0007938.s002.tif]

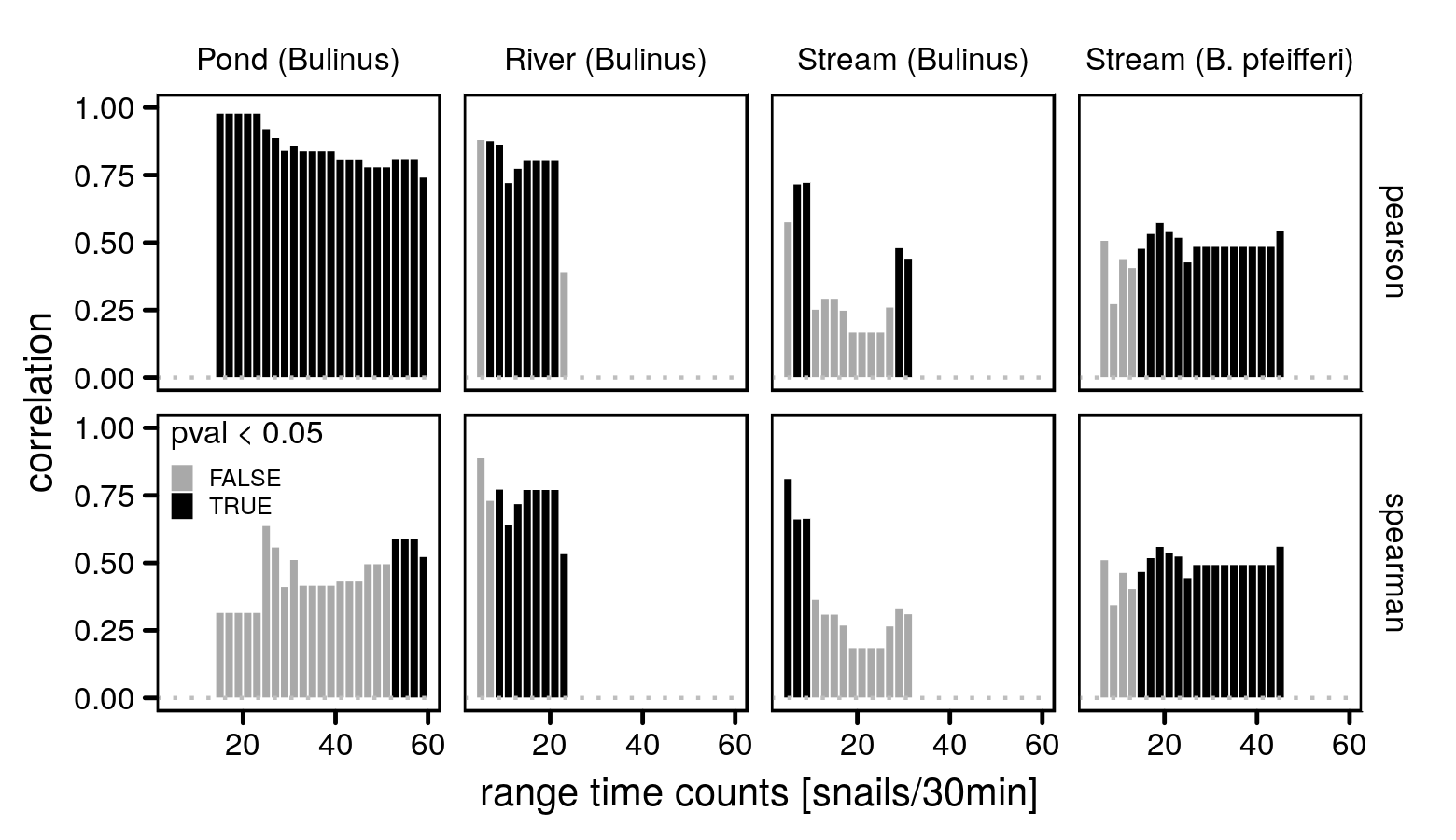

Supplement: S2 Fig — Pearson (top row) and Spearman (bottom row) correlations computed for increasing ranges of time-based mean counts. (TIF) [file pntd.0007938.s003.tif]

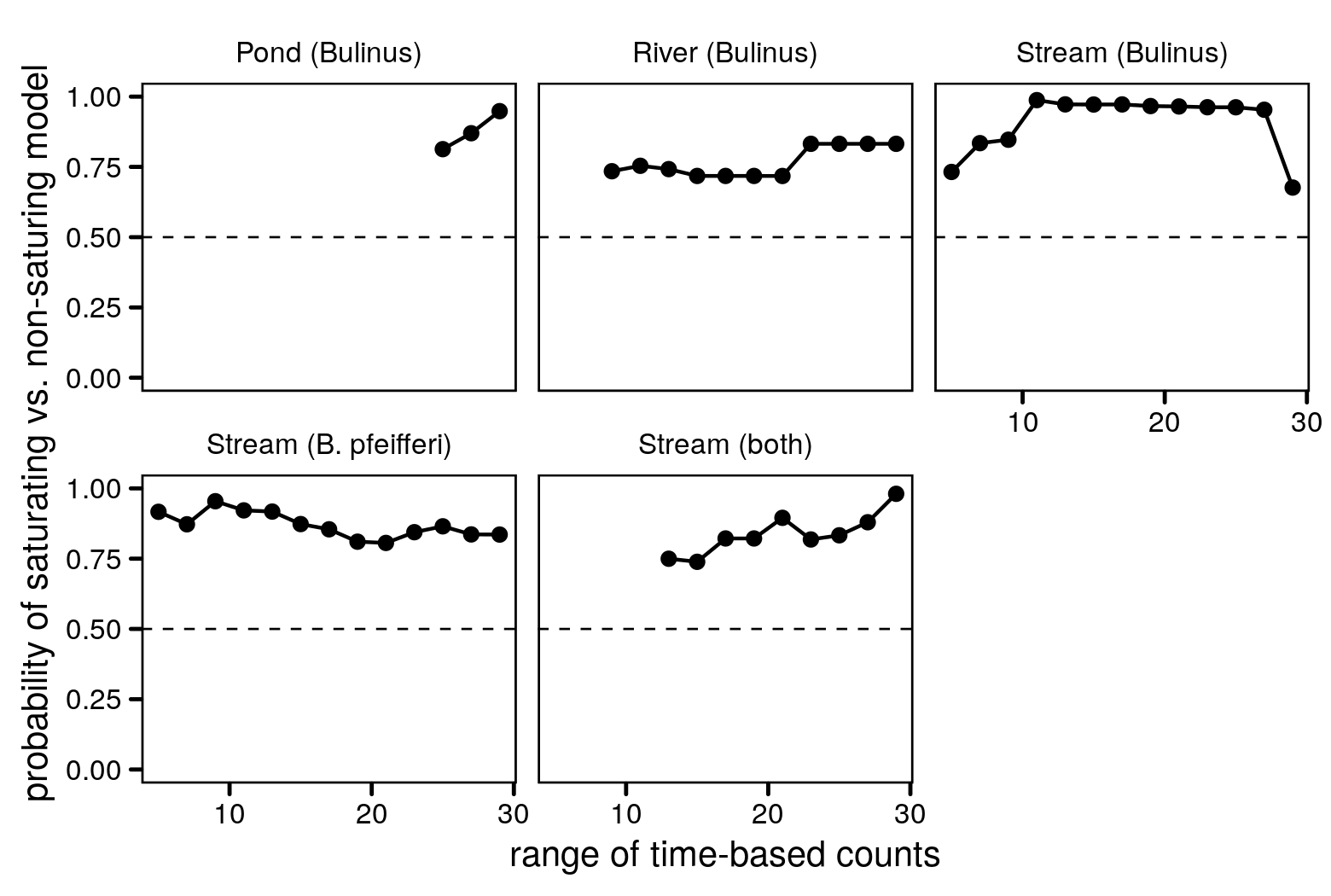

Supplement: S3 Fig — Support was computed as in S2 Table. Each point corresponds to the support of saturating vs. non-saturating models when considering only sampling data within the range of time-based counts. The absence of points bellow 20 snails/30min are due to the absence of data. (TIF) [file pntd.0007938.s004.tif]

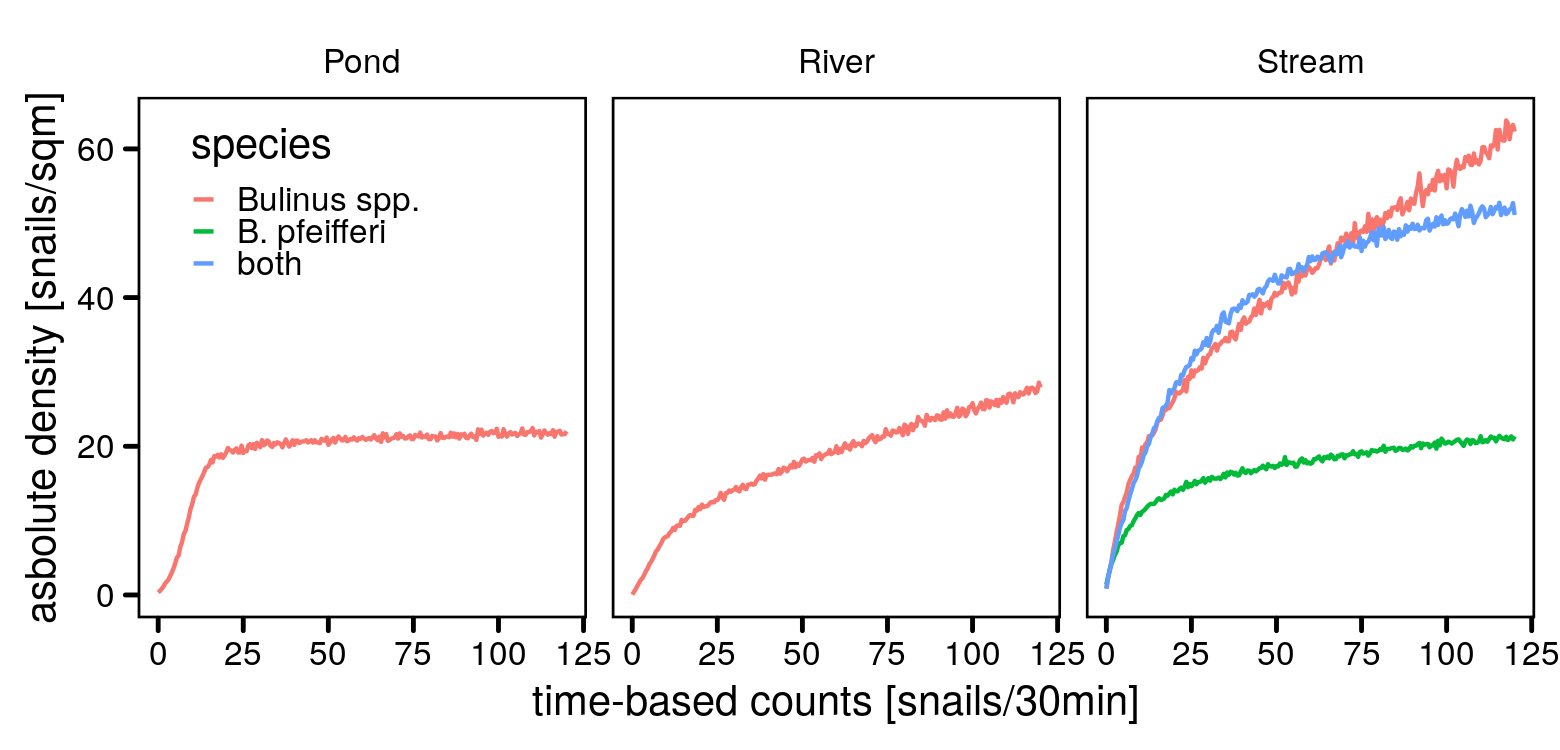

Supplement: S4 Fig — Simulations were performed using the 95% credible model set (S1 Table) for each species-habitat configuration. Simulations are shown in terms of the mean (lines) for 5000 simulations per time-based count. (TIF) [file pntd.0007938.s005.tif]
