# Supplementary material for: Comparative analysis of time-based and quadrat sampling in seasonal population dynamics of intermediate hosts of human schistosomes
Source: PLoS Negl Trop Dis. 2019 Dec 20;13(12):e0007938. doi: 10.1371/journal.pntd.0007938 (PMC6957212; doi:10.1371/journal.pntd.0007938)
Supplement: S1 Table — (PDF) [file pntd.0007938.s006.pdf]

Species	Habitat	Transfer	Measurement	σ	l	n	AICc	w_i	$\sum_{k=1}^{i} w_k$
Bulinus spp.	River	monoMolecular	zip	constant	113.29	3	232.77	0.12	0.12
		michaelisMenten	zip	constant	113.36	3	232.92	0.11	0.24
		gompertz	zip	constant	112.48	4	233.29	0.09	0.33
		linear	zip	constant	113.65	3	233.49	0.09	0.42
		logistic	zip	constant	112.76	4	233.85	0.07	0.49
		monoMolecular	zinb	constant	113.29	4	234.90	0.04	0.53
		monoMolecular	zip	logistic	113.29	4	234.90	0.04	0.57
		michaelisMenten	zinb	constant	113.36	4	235.05	0.04	0.61
		michaelisMenten	zip	logistic	113.36	4	235.05	0.04	0.65
		gompertz	zinb	constant	112.48	5	235.46	0.03	0.69
		gompertz	zip	logistic	112.48	5	235.46	0.03	0.72
		monoMolecular	negbin	-	114.70	3	235.59	0.03	0.75
		linear	zip	logistic	113.65	4	235.62	0.03	0.78
		linear	zinb	constant	113.65	4	235.62	0.03	0.81
		michaelisMenten	negbin	-	114.87	3	235.93	0.03	0.83
		logistic	zinb	constant	112.76	5	236.01	0.02	0.86
		logistic	zip	logistic	112.76	5	236.01	0.02	0.88
		gompertz	negbin	-	113.89	4	236.10	0.02	0.90
		logistic	negbin	-	114.19	4	236.70	0.02	0.92
		monoMolecular	zinb	logistic	113.29	5	237.07	0.01	0.94
		michaelisMenten	zinb	logistic	113.36	5	237.22	0.01	0.95
Bulinus spp.	Pond	logistic	zip	constant	188.26	4	384.84	0.31	0.31
		gompertz	zip	constant	189.05	4	386.43	0.14	0.45
		logistic	zip	logistic	188.20	5	386.89	0.11	0.56
		logistic	zinb	constant	188.26	5	387.01	0.10	0.66
		monoMolecular	zip	constant	191.09	3	388.38	0.05	0.71
		gompertz	zip	logistic	188.96	5	388.40	0.05	0.77
		gompertz	zinb	constant	189.05	5	388.60	0.05	0.81
		logistic	zinb	logistic	188.25	6	389.19	0.03	0.85
		monoMolecular	zip	logistic	190.55	4	389.43	0.03	0.88
		michaelisMenten	zip	constant	191.65	3	389.49	0.03	0.91
		monoMolecular	zinb	constant	191.09	4	390.51	0.02	0.93
		michaelisMenten	zip	logistic	191.13	4	390.59	0.02	0.94
B. pfeifferi	Stream	michaelisMenten	zip	constant	358.18	3	722.44	0.19	0.19
		michaelisMenten	zip	logistic	357.61	4	723.37	0.12	0.32
		monoMolecular	zip	constant	358.87	3	723.83	0.10	0.41
		michaelisMenten	zinb	constant	358.18	4	724.50	0.07	0.48
		monoMolecular	zip	logistic	358.31	4	724.77	0.06	0.54
		gompertz	zip	constant	358.42	4	724.99	0.05	0.60
		logistic	zip	constant	358.57	4	725.30	0.05	0.64
		michaelisMenten	zinb	logistic	357.61	5	725.45	0.04	0.69
		monoMolecular	zinb	constant	358.87	4	725.88	0.03	0.72
		gompertz	zip	logistic	357.84	5	725.90	0.03	0.76
		logistic	zip	logistic	357.94	5	726.10	0.03	0.79
		linear	zip	constant	360.35	3	726.80	0.02	0.81
		linear	zip	logistic	359.33	4	726.81	0.02	0.83
		monoMolecular	zinb	logistic	358.31	5	726.85	0.02	0.85
		gompertz	zinb	constant	358.42	5	727.06	0.02	0.87
		logistic	zinb	constant	358.57	5	727.37	0.02	0.89
		linear	hup	logistic	359.64	4	727.44	0.02	0.90
		michaelisMenten	hup	logistic	359.89	4	727.93	0.01	0.92
		gompertz	zinb	logistic	357.84	6	727.99	0.01	0.93
		logistic	zinb	logistic	357.94	6	728.19	0.01	0.94
		logistic	hup	logistic	359.16	5	728.55	0.01	0.95
		gompertz	hup	logistic	359.18	5	728.59	0.01	0.96
Bulinus spp.	Stream	michaelisMenten	negbin	-	460.71	3	927.52	0.22	0.22
		linear	zinb	logistic	458.68	5	927.58	0.21	0.43
		monoMolecular	negbin	-	461.27	3	928.62	0.13	0.55
		michaelisMenten	zinb	constant	460.53	4	929.21	0.09	0.65
		linear	negbin	-	461.94	3	929.98	0.06	0.71
		monoMolecular	zinb	constant	461.12	4	930.38	0.05	0.76
		logistic	zinb	logistic	459.10	6	930.51	0.05	0.81
		gompertz	zinb	logistic	459.12	6	930.55	0.05	0.86
		michaelisMenten	zinb	logistic	460.38	5	930.99	0.04	0.90
		linear	zinb	constant	461.62	4	931.39	0.03	0.93
		monoMolecular	zinb	logistic	461.12	5	932.46	0.02	0.95
both	Stream	michaelisMenten	zinb	constant	552.43	4	1113.01	0.19	0.19
		michaelisMenten	negbin	-	553.60	3	1113.30	0.17	0.36
		monoMolecular	zinb	constant	552.73	4	1113.61	0.14	0.51
		monoMolecular	negbin	-	553.92	3	1113.93	0.12	0.63
		michaelisMenten	zinb	logistic	552.14	5	1114.50	0.09	0.72
		monoMolecular	zinb	logistic	552.47	5	1115.17	0.07	0.79
		michaelisMenten	hunb	logistic	553.26	5	1116.74	0.03	0.82
		monoMolecular	hunb	logistic	553.42	5	1117.07	0.03	0.84
		gompertz	zinb	constant	553.47	5	1117.16	0.02	0.87
		linear	negbin	-	555.56	3	1117.21	0.02	0.89
		gompertz	negbin	-	554.62	4	1117.39	0.02	0.91
		linear	zinb	constant	554.72	4	1117.59	0.02	0.93
		logistic	zinb	constant	554.14	5	1118.50	0.01	0.94
		gompertz	zinb	logistic	553.13	6	1118.59	0.01	0.96
