# Supplementary material for: Comparative analysis of time-based and quadrat sampling in seasonal population dynamics of intermediate hosts of human schistosomes
Source: PLoS Negl Trop Dis. 2019 Dec 20;13(12):e0007938. doi: 10.1371/journal.pntd.0007938 (PMC6957212; doi:10.1371/journal.pntd.0007938)
Supplement: S2 Table — Unconditional mean and 95% confidence intervals (in parenthesis). Note that these values do not correspond to absolute densities which require in addition the accounting for zero-inflation in counts. (PDF) [file pntd.0007938.s007.pdf]

Species	Habitat	snails/quadrat	snails/m^2
Bulinus	Pond	2.70 (2.06-3.53)	29.95 (22.86-39.23)
Bulinus	River	7.17 (0.00-13030.09)	79.72 (0.04-144778.76)
Biomphalaria	Stream	2.14 (1.37-3.36)	23.81 (15.17-37.38)
Bulinus	Stream	4.47 (2.45-8.13)	49.62 (27.26-90.34)
both	Stream	2.46 (1.05-5.75)	27.28 (11.65-63.91)
