# Supplementary material for: Comparative analysis of time-based and quadrat sampling in seasonal population dynamics of intermediate hosts of human schistosomes
Source: PLoS Negl Trop Dis. 2019 Dec 20;13(12):e0007938. doi: 10.1371/journal.pntd.0007938 (PMC6957212; doi:10.1371/journal.pntd.0007938)
Supplement: S3 Table — Unconditional mean and 95% confidence intervals (in parenthesis). (PDF) [file pntd.0007938.s008.pdf]

Species	Habitat	σ
Bulinus	Pond	0.38 (0.28-0.49)
Bulinus	River	0.41 (0.05-0.90)
Biomphalaria	Stream	0.42 (0.33-0.51)
Bulinus	Stream	0.24 (0.00-1.00)
both	Stream	0.08 (0.00-1.00)
